# Tetra-*n*-butyl­ammonium orotate monohydrate: knowledge-based comparison of the results of accurate and lower-resolution analyses and a non-routine disorder refinement

**DOI:** 10.1107/S2056989019013380

**Published:** 2019-10-08

**Authors:** Irene Ara, Zeineb Basdouri, Larry R. Falvello, Mohsen Graia, Pablo Guerra, Milagros Tomás

**Affiliations:** a Instituto de Síntesis Quimica y Catálisis Homogénea (ISQCH), C.S.I.C.-University of Zaragoza, Departamento de Química Inorgánica, Pedro Cerbuna 12, E-50009 Zaragoza, Spain; b Laboratoire de Matériaux, Cristallochimie et Thermodynamique Appliquée, Département de Chimie, Faculté des Sciences de Tunis, Université de Tunis El Manar, 2092 El Manar II, Tunis, Tunisia; c University of Zaragoza-C.S.I.C., Instituto de Ciencia de Materiales de Aragón (ICMA), Departamento de Química Inorgánica, E-50009 Zaragoza, Spain; d Université de Sfax, Faculté de Sciences de Sfax, Route de la Soukra Km 4, Sfax 3038, Tunisia

**Keywords:** crystal structure, orotate, hydro­phobic–hydro­philic mol­ecular ion pair, Hirshfeld surface analysis, knowledge-based analysis, *Mogul* geometry check

## Abstract

The title hydrated mol­ecular salt (systematic name: tetra-*n*-butyl­ammonium 2,6-dioxo-1,2,3,6-tetra­hydro­pyrimidine-4-carboxyl­ate monohydrate), C_16_H_36_N^+^·C_5_H_3_N_2_O_4_^−^·H_2_O, crystallizes with N—H⋯O and O—H⋯O hydrogen-bonded double-stranded anti­parallel ribbons consisting of the hydro­philic orotate monoanions and water mol­ecules, separated by the bulky hydro­phobic cations. The hydro­phobic and hydro­philic regions of the structure are joined by weaker non-classical C—H⋯O hydrogen bonds. An accurate structure analysis conducted at *T* = 100 K is compared to a lower-resolution less accurate determination using data measured at *T* = 295 K. The results of both analyses are evaluated using a knowledge-based approach, and it is found that the less accurate room-temperature structure analysis provides geometric data that are similar to those derived from the accurate low-temperature analysis, with both sets of results consistent with previously analyzed structures. A minor disorder of one methyl group in the cation at low temperature was found to be slightly more complex at room temperature; while still involving a minor fraction of the structure, the disorder at room temperature was found to require a non-routine treatment, which is described in detail.

## Abstract 2

The analysis of a hydro­phobic–hydro­philic ion-pair structure at two temperatures permits the comparative evaluation of the accurate geometric results obtained at low temperature and less accurate results at room temperature using a knowledge-based approach.

## Chemical context   

We report here the structure analysis at two temperatures (**1** at 100 K and **2** at 295 K) of an organic salt formed by a bulky, hydro­phobic cation, ^*n*^Bu_4_N^+^, and the compact, hydro­philic anion C_5_H_3_N_2_O_4_^−^, formed by single deprotonation of orotic acid. Crystals of this material are monohydrated, and the water mol­ecule plays an integral role in the structure.
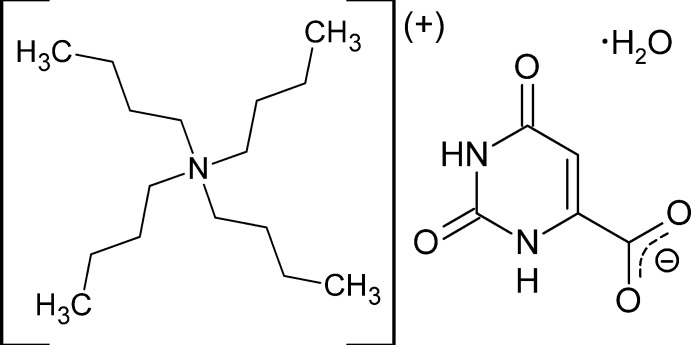


Orotic acid, 2,4-dioxo-1*H*-pyrimidine-6-carb­oxy­lic acid, C_5_H_4_N_2_O_4_, is important in a multitude of roles in biochemistry, among them as a precursor in the synthesis of uridine monophosphate (UMP) and thus of the pyrimidine nucleotides (Löffler *et al.*, 2015 ▸, 2016 ▸, 2018 ▸).

Our own inter­est in orotic acid, and in the conjugate bases formed by single and double deprotonation of orotic acid, arises from the functional groups that they present to their surroundings, which endow them with the ability to bind to a transition metal while at the same time forming energetically significant, directed and possibly structure-directing, inter­actions with their environment in a crystal. We have encountered, for example, a system in which stereoisomer selection for a six-coordinate Ni^II^ complex is achieved by enabling or vitiating hydrogen-bond formation in crystals of the product (Falvello *et al.*, 2007 ▸). In another study (Castro *et al.*, 2017 ▸), it was found that the ^*n*^Bu_4_N^+^ salt of a Co^III^ orotate complex, namely (^*n*^Bu_4_N)[Co(orot)_2_(bipy)]·3H_2_O, undergoes an order–disorder phase transition, which upon recycling and repeating suffers arrest, which leaves the sample in a two-domain, two-structure form (monoclinic/triclinic).

It is in the context of phase transitions that we find the simple cation tetra-*n*-butyl­ammonium, ^*n*^Bu_4_N^+^ or C_16_H_36_N^+^, to be of inter­est. It is known that the presence of even a single *n*-butyl group can be sufficient to incite an order–disorder transition when, for example, the temperature is varied (Willett *et al.*, 2005 ▸).

While our inter­est in orotic acid and orotates stems from their utility in coordination chemistry, it is also pertinent to explore mol­ecular solids in which these fragments are present without metals. To date, six unique crystal structures have been analyzed of solids containing orotic acid in the absence of coordination compounds (ten analyses, including duplicates); and three analyses have been reported with orotic acid co-crystallized with orotate complexes of Co, Pr and Nd. Singly deprotonated orotate – Horot^−^, deprotonated at the carboxyl­ate function – figures in some 46 previously reported structure analyses, 16 of which also have *d*-block elements and six of which are lanthanoid compounds. There is also one structure of a uranium complex of Horot^−^. Some 15 Horot^−^-containing structures have no metal atom present.

With this as background, we undertook the structure analysis of the monohydrate of tetra-*n*-butyl­ammonium 2,4-dioxo-1*H*-pyrimidine-6-carboxyl­ate, (^*n*^Bu_4_N)(C_5_H_3_N_2_O_4_), at room temperature and at 100 K, to establish the structural organization adopted by this hydro­phobic–hydro­philic ion pair and to explore the possibility of an order–disorder phase transition, as is seen with some regularity in *n-*butyl-containing mol­ecular crystals.

## Structural commentary   

One of the motivations for this study was to observe the packing pattern adopted by a bulky hydro­phobic cation and a compact hydro­philic anion when crystallized together. In the event, there are no solvent-accessible voids, as calculated by *PLATON* (Spek, 2009 ▸); however, this full packing arrangement is achieved with the incorporation of one water mol­ecule per formula unit. Packing and scattering are more efficient at low temperature; we will discuss the structure first with reference to the analysis at *T* = 100 K; some comparisons between the two analyses will be presented at the end.

**Figure 1:**
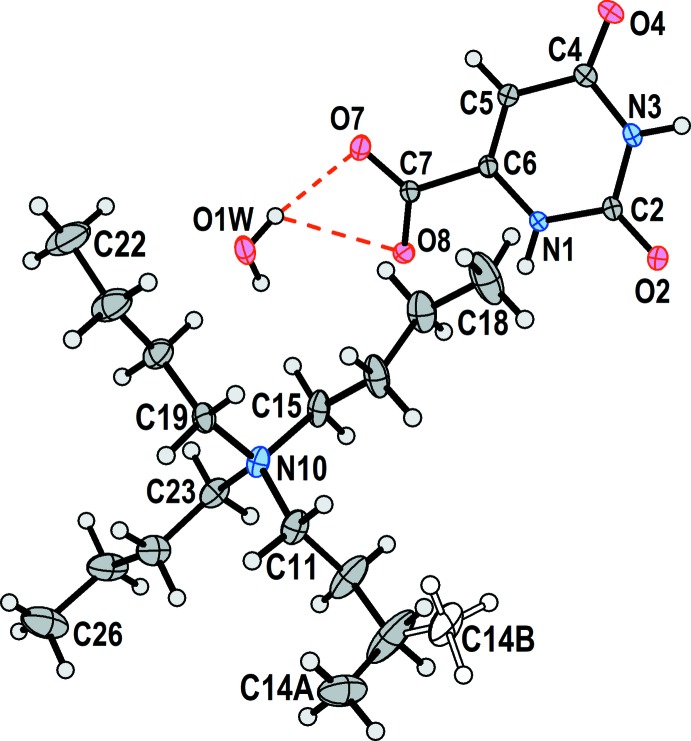
The asymmetric unit of **1** at 100 K. Non-hydrogen atoms are represented by their 50% probability displacement ellipsoids. Dashed red lines represent hydrogen bonds. C14*A* and C14*B* are the major and minor components of the disordered methyl group.

**Figure 2:**
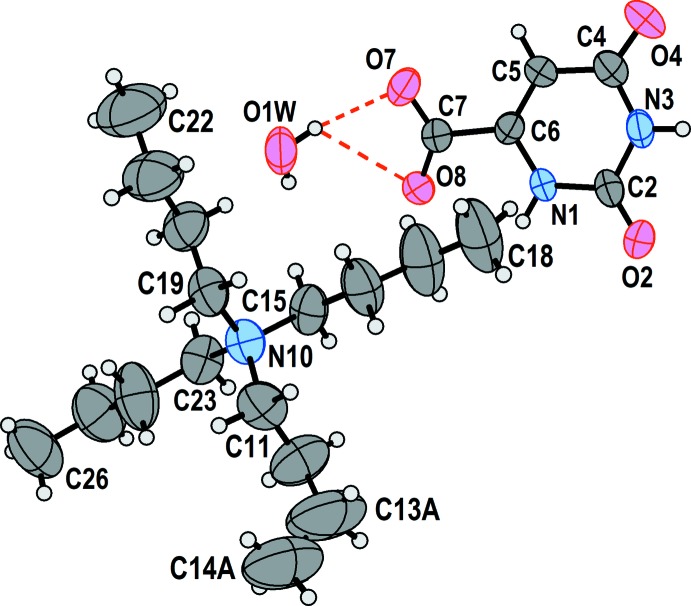
The asymmetric unit of **2** at 295 K. Non-hydrogen atoms are represented by their 50% probability displacement ellipsoids. Dashed red lines represent hydrogen bonds. C13*A* and C14*A* represent one component of a disordered ethyl fragment, whose other components are not shown.

Displacement ellipsoid plots of the two structures are shown in Fig. 1 ▸ (100 K) and Fig. 2 ▸ (295 K). The two drawings have the same scale, and it is clear that, as expected, the lower-temperature structure has notably reduced displacement as compared to the structure at room temperature.

### Supra­molecular features   

**Figure 3:**
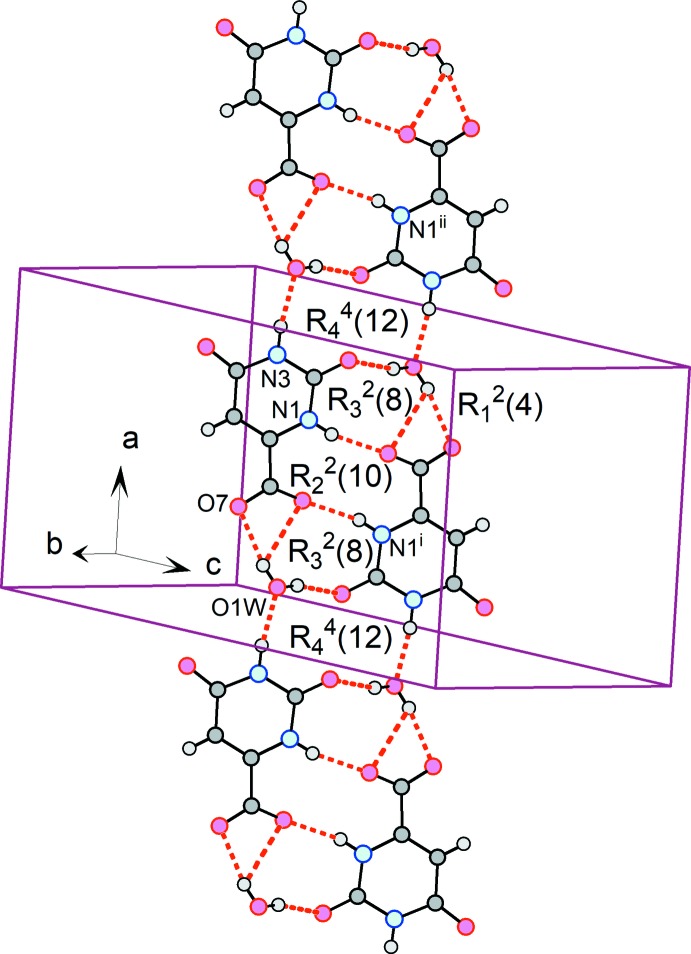
The ladder-like chain formed by the hydro­philic fragments, from the structure at *T* = 100 K. [Symmetry codes: (i) −*x* + 1, −*y* + 1, −*z* + 1; (ii) −*x* + 2, −*y* + 1, −*z* + 1.]

The structure is segregated into hydro­philic and hydro­phobic zones. Firstly, a network of N—H⋯O and O—H⋯O hydrogen bonds link the Horot^−^ anions and water mol­ecules into a ladder-like chain propagating in the *a*-axis direction and lying in the (011) plane (Table 1 ▸ for *T* = 100 and Table 2 ▸ for *T* = 295 K; Fig. 3 ▸). Four different types of hydrogen-bonded rings form an uninter­rupted fused-ring system along the length of this chain. Symmetry relatives of the 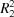
(10) ring at the center of the segment shown in Fig. 3 ▸ occupy inversion centers at (1/2 + *n*, 1/2, 1/2), where *n* is an integer. The chain is further propagated through an 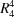
(12) ring whose congeners are on inversion centers at (*n*, 1/2, 1/2), with *n* an integer. The components of this chain are related by the 2_1_ screw axis and the *c*-glide to the constituent fragments of chains – also parallel to the *a* axis of the cell but lying in (01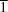) planes – passing through centers of inversion at (0, 0, 0), (1/2, 0, 0) and lattice-related positions.

**Figure 4:**
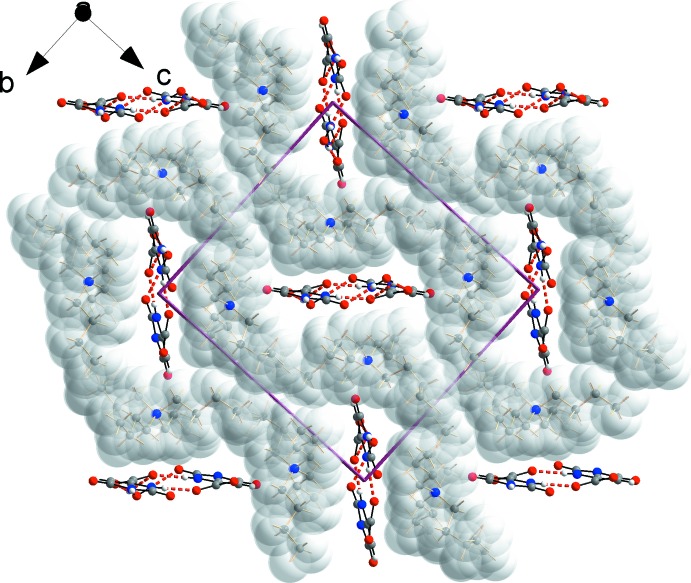
Packing of the ^*n*^Bu_4_N^+^ cations and Horot^(-)^–H_2_O chains in **1**. Dashed red lines represent hydrogen bonds within the hydro­philic zones. The H atoms of the cations are represented by spheres with the van der Waals radius of 1.2 Å.

The hydro­phobic cations surround the orotate–water chains, filling in the remaining space in the structure (Fig. 4 ▸). That the cell is efficiently filled can be seen in the Kitaigorodsky packing indices (KPI: percent filled space; Kitaigorodsky, 1973 ▸) of 66.8 for **1** and 63.2 for **2**. [In order to perform the calculation of the KPI using *PLATON* (Spek, 2009 ▸) it was necessary to create a structure model with only the principal components of disorder present.] For comparison purposes, we note that a structure consisting of close-packed spheres fills 74.0% of its FCC unit cell.

**Figure 5:**
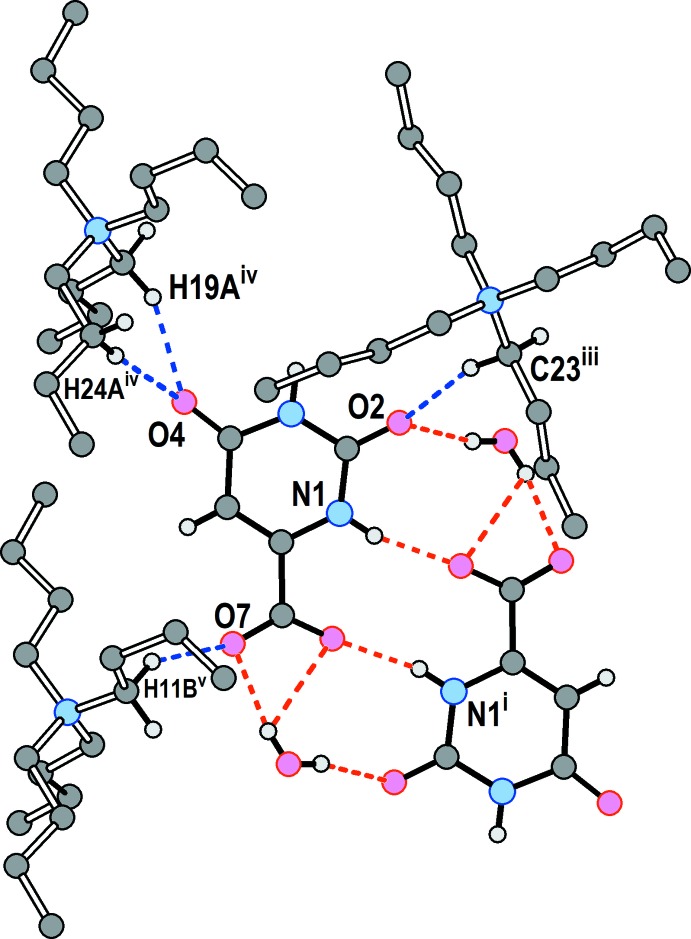
Partial view of the packing in **1**, showing hydrogen bonds within the hydro­philic chain (red dashed lines) and non-classical C—H⋯O hydrogen bonds (blue dashed lines) between methyl­ene H atoms of the cations and oxygen atoms of the orotate anions. [Symmetry codes: (i) −*x* + 1, −*y* + 1, −*z* + 1; (iii) *x* + 1, *y*, *z*; (iv): *x* + 1, −*y* + 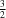, *z* − 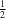; (v) *x*, −*y* + 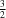, *z* − 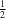.] Butyl-group H atoms not involved in hydrogen bonds have been omitted. The minor congener of the terminal methyl group has been omitted.

The hydro­phobic and hydro­philic sectors of the structure are not strictly separated, as there are favorable inter­actions between them (Table 1 ▸, Fig. 5 ▸). Each orotate anion accepts a total of four non-classical hydrogen bonds from the methyl­ene groups of three surrounding ^*n*^Bu_4_N^(+)^ cations. Fig. 5 ▸ shows a segment of the hydro­philic chain, with its hydrogen bonds in red, and three neighboring cations with the C—H⋯O inter­actions in blue. The flexibility of the butyl groups along with their capacity for forming directed inter­actions with the anions and dispersion-based inter­actions among themselves, is key to the ability of this material to form well-packed crystals.

**Figure 6:**
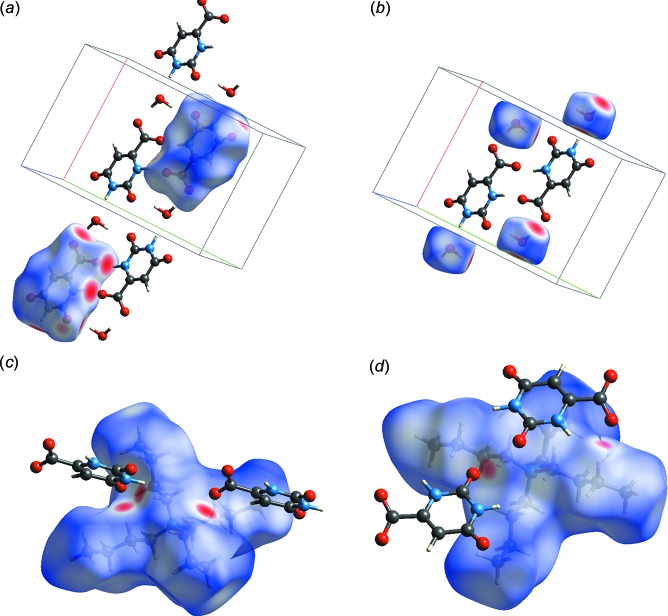
Hirshfeld surfaces based on *d_norm_* for (*a*) the orotate anion, seen from two viewpoints in the anti­parallel chains; (*b*) the water mol­ecule, seen from two angles; (*c*) and (*d*) the cation, viewed from two opposite sides.

The overall layout of the structure and the inter­actions that consolidate it are summarized graphically in the Hirshfeld surfaces (Spackman & Jayatilaka, 2009 ▸), which are presented here only for the low-temperature determination (Fig. 6 ▸). Fig. 6 ▸ *a* shows the Hirshfeld surface for the anion from two viewpoints, within its crystalline surroundings. It is clear that its principal inter­actions lie within the hydro­philic zone. Fig. 6 ▸ *b* rounds out the picture, showing through the Hirshfeld surfaces that the major inter­actions of the water mol­ecules also lie within the hydro­philic sector of the structure. Fig. 6 ▸ *c* and 6*d* show the Hirshfeld surface of the cation from two opposite view directions. The scarce inter­actions consist of two non-classical hydrogen bonds on each side. Fingerprint calculations reveal that the close H(inter­nal)⋯O(external) inter­actions account for only 14.1% of the points on the surface. These can be compared to H(*i*)⋯O(*e*) and O(*i*)⋯H(*e*) values of 27.5% and 32.3%, respectively, for the water mol­ecule and 6.2% and 49.2% for the orotate anion.

## Database survey: knowledge-based comparison of the analyses at two temperatures   

The presence of bulky aliphatic groups clearly influences the diffraction from these crystals. The structure at room temperature suffers not only from a more complex disorder of one terminal ethyl group, but also produces weak diffraction, to the extent that from intensity statistics we estimate the effective resolution of the data to be about 1.0 Å. The data at *T* = 100 K are much stronger and give what in present times is regarded as an accurate result, which includes the observation of positive difference density at the centers of most of the bonds not involving H atoms.

It is thus instructive to compare the geometric parameters derived from the two analyses.

**Figure 7:**
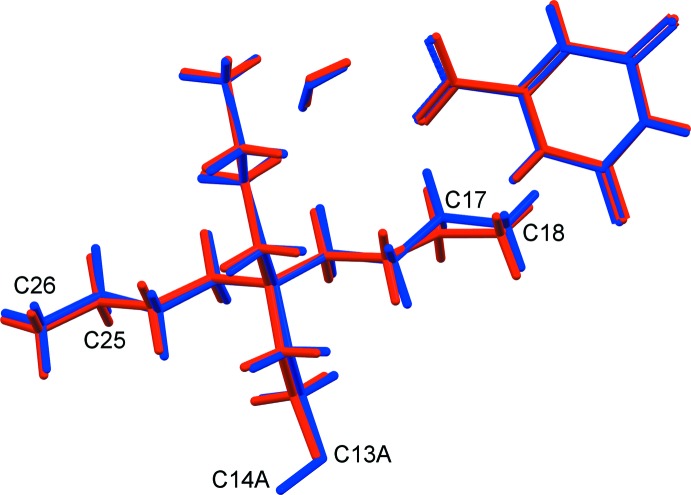
Overlay of the asymmetric units for the analyses at *T* = 100 K (blue) and *T* = 295 K (red).

An overlay of all corresponding non-H atoms in the two structures, excluding the disordered Et fragment at C13 and C14, gives an r.m.s. deviation of 0.144 Å. As can be seen in Fig. 7 ▸, most of the deviation resides in the slightly different conformations of two of the terminal Et groups of the cation, namely C17/C18 (0.35, 0.21 Å deviation for C17 and C18, respectively) and C25/C26 (0.17, 0.18 Å for C25, C26, respectively).

### *Mogul* geometry check   

Extending the geometric comparisons to the possible differences between these two determinations, on one hand, and prior analyses involving similar chemical fragments, on the other, we performed a *Mogul* geometry check, in which bond lengths and bond angles found in these structures are compared to those of fragments of the same chemical nature found in the CSD (Cambridge Structural Database; Groom *et al.*, 2016 ▸). All results are compiled in the supporting information. Briefly, there are no gross outliers in these two analyses; however, the area of the carboxyl­ate group and its linkage to the ring of the anion shows an inter­esting trend in both analyses. A relevant fact in this regard is that the dihedral angle between the orotate ring and the pendant carboxyl­ate group is 23.14 (8)° at *T* = 100 K and 20.4 (2)° at room temperature. While the *Mogul* geometry check does not encounter any important outliers for either analysis, it does signal some slightly larger deviations from previous results, in the conjugated region where the ring and carboxyl­ate group are joined, in keeping with the torsion angle that reduces π–π overlap between C6 and C7. Thus, considering the mean and standard deviation σ of the bond distances found in previous structure analyses with chemically similar groups, at low temperature the two nominally delocalized C⋯O bonds C7—O7 and C7—O8 are 0.734 σ and 1.620 σ shorter than the mean; C5—C6 is 1.411 σ shorter; and C6—C7 is 1.985 σ longer. For *T* = 295 K the analogous deviations are 3.047 and 4.820 σ for C7—O7 and C7—O8, 3.065 σ for C5—C6 and 1.665 σ for C6—C7 – all in the expected direction from the mean. These variations are not extreme and might be taken as barely significant statistically. However, we consider it noteworthy that they stand out in comparison with the analogous values for the rest of the structure, and that similar results are obtained at both temperatures (Tables S1 and S2 in the supporting information).

If we consider the reported structures of Horot^−^ with alkali counter-ions, the Horot fragments in the anhydrous K^+^ and Rb^+^ salts (both: Bekiroglu & Kristiansson, 2002 ▸; K^+^: Clegg & Nichol, 2018*a* ▸; Rb^+^: Martínez *et al.*, 2008 ▸) are co-planar. However, for the hydrated compounds they are not coplanar, although the angles are smaller than in the NBu_4_^+^ compound. For K(Horot)·H_2_O the analogue of the O—C—C6—N1 torsion angle is −9.59° (CSD refcode MIJLUN, Yeşilel *et al.*, 2007 ▸); and for three analyses of Li(Horot)·H_2_O, smaller values were found for the analogous torsion angle: SIMZOD 3.07° (Bach *et al.*, 1990 ▸); SIMZOD01 3.83°, (Lutz, 2001 ▸); SIMZOD02 3.82° (Clegg & Nichol, 2018*b* ▸).

## Synthesis and crystallization   

2 ml of a 1.5 *M* aqueous solution of NBu_4_OH (3 mmol) was added to a suspension of 0.7 g (4 mmol) of orotic acid monohydrate, H_2_Orot·H_2_O, in 2 ml of water. The suspension was stirred for 3 h at room temperature and then filtered through paper in order to remove the excess of H_2_Orot·H_2_O. Partial evaporation of the solution at 303 K produced colorless crystals of [NBu_4_][HOrot]·H_2_O, which were removed from the solution and dried with paper (0.643 g, 49.5% yield).

## Refinement   

Crystal data, data collection parameters and structure refinement residuals are given in Table 3 ▸. Single-crystal diffraction data were gathered from two crystals, one at *T* = 100 K, **1**, and the other at room temperature, **2**. The structure was solved *ab initio* from each of the two data sets using iterative methods (*SHELXT* 2014/5; Sheldrick, 2015*a* ▸) and refined using full-matrix least-squares analysis (*SHELXL2018/1*; Sheldrick, 2015*b* ▸). For **1**, one of the *n-*Bu groups of the cation, namely C11–C14, had its terminal CH_3_ group disordered over two sets of sites, whose occupancy ratio was refined to 0.698 (4)/0.302 (4). For **2**, measured at room temperature, the same *n-*Bu group suffered a more complex disorder, with the γ*-*C atom, C13, disordered over two positions and the δ*-*C atom, C14, disordered over three positions. This disorder assembly was inter­preted as being composed of four disorder groups; the structure model was composed and refined so as to produce chemically sound stoichiometry for the individual disorder groups and for the assembly as a whole. The inter­ested reader is referred to the supporting information and the embedded, commented instruction file in the CIF for full details. The H atoms of methyl­ene groups in both structures were placed at idealized positions and refined as riding atoms. The H atoms of all methyl groups in **1** and of the ordered methyl groups in **2** were placed at positions derived from local Fourier calculations and permitted to rotate but not tilt in the refinement. The H atoms of disordered CH_3_ groups in **2** were placed at positions calculated to give staggered conformations about the local C—C bond and refined as riding atoms. For CH_2_ groups, *U*_iso_(H) were set to 1.2*U*_eq_ of their respective bonding partners. For CH_3_, *U*_iso_(H) were set to 1.5*U*_eq_(C). The H atoms of the orotate anion and the water mol­ecule were located in difference Fourier maps for both analyses; their positions were refined freely and their *U*_iso_ were refined freely for **1** and set to 1.2*U*_eq_ of their respective bonding partners for **2**.

## Supplementary Material

Crystal structure: contains datablock(s) 100K, 295K, global. DOI: 10.1107/S2056989019013380/hb7858sup1.cif

Structure factors: contains datablock(s) 100K. DOI: 10.1107/S2056989019013380/hb7858100Ksup2.hkl

Structure factors: contains datablock(s) 295K. DOI: 10.1107/S2056989019013380/hb7858295Ksup3.hkl

Supporting information file. DOI: 10.1107/S2056989019013380/hb7858100Ksup4.cml

Supporting information file. DOI: 10.1107/S2056989019013380/hb7858sup5.pdf

CCDC references: 1956837, 1956838, 1956837, 1956838

Additional supporting information:  crystallographic information; 3D view; checkCIF report

## Figures and Tables

**Table 1:** Hydrogen-bond geometry (Å, °) for **1** [Chem scheme1]

*D*—H⋯*A*	*D*—H	H⋯*A*	*D*⋯*A*	*D*—H⋯*A*
N1—H1⋯O8^i^	0.860 (16)	1.924 (16)	2.7668 (12)	166.4 (14)
N3—H3⋯O1*W* ^ii^	0.901 (17)	1.913 (17)	2.8081 (12)	171.8 (15)
C11—H11*B*⋯O7^iii^	0.99	2.25	3.1462 (15)	151
C19—H19*A*⋯O4^iv^	0.99	2.28	3.1878 (14)	151
C23—H23*A*⋯O2^v^	0.99	2.37	3.3305 (14)	164
C24—H24*A*⋯O4^iv^	0.99	2.34	3.3197 (19)	171
O1*W*—H1*WA*⋯O7	0.85 (2)	2.00 (2)	2.8396 (12)	169.2 (18)
O1*W*—H1*WA*⋯O8	0.85 (2)	2.64 (2)	3.1155 (12)	117.3 (15)
O1*W*—H1*WB*⋯O2^i^	0.87 (2)	2.01 (2)	2.8618 (13)	168.3 (19)

Symmetry codes: (i) 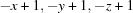; (ii) 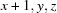; (iii) 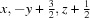; (iv) 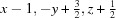; (v) 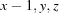.

**Table 2:** Hydrogen-bond geometry (Å, °) for **2** [Chem scheme1]

*D*—H⋯*A*	*D*—H	H⋯*A*	*D*⋯*A*	*D*—H⋯*A*
N1—H1⋯O8^i^	0.866 (19)	1.96 (2)	2.800 (3)	162.1 (18)
N3—H3⋯O1*W* ^ii^	0.86 (2)	1.96 (2)	2.807 (2)	170 (2)
C11—H11*A*⋯O7^iii^	0.97	2.26	3.168 (3)	155
C19—H19*A*⋯O4^iv^	0.97	2.28	3.226 (3)	164
C23—H23*B*⋯O2^v^	0.97	2.44	3.386 (3)	166
C24—H24*B*⋯O4^iv^	0.97	2.47	3.346 (4)	151
O1*W*—H1*WA*⋯O7	0.85 (2)	2.03 (2)	2.874 (2)	172 (2)
O1*W*—H1*WA*⋯O8	0.85 (2)	2.59 (3)	3.074 (2)	118 (2)
O1*W*—H1*WB*⋯O2^i^	0.76 (3)	2.15 (3)	2.895 (2)	164 (3)

Symmetry codes: (i) 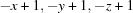; (ii) 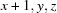; (iii) 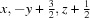; (iv) 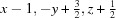; (v) 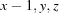.

**Table 3:** Experimental details

	**1**	**2**
Crystal data		
Chemical formula	C_16_H_36_N^+^·C_5_H_3_N_2_O_4_^−^·H_2_O	C_16_H_36_N^+^·C_5_H_3_N_2_O_4_^−^·H_2_O
*M*_r_	415.57	415.57
Crystal system, space group	Monoclinic, *P*2_1_/*c*	Monoclinic, *P*2_1_/*c*
Temperature (K)	100	295
*a*, *b*, *c* (Å)	10.0905 (5), 14.8664 (8), 16.1261 (9)	10.1335 (5), 14.6690 (7), 16.9205 (8)
β (°)	97.347 (5)	96.630 (4)
*V* (Å^3^)	2399.2 (2)	2498.4 (2)
*Z*	4	4
Radiation type	Mo *K*α	Mo *K*α
μ (mm^−1^)	0.08	0.08
Crystal size (mm)	0.31 × 0.18 × 0.16	0.55 × 0.23 × 0.09

Data collection		
Diffractometer	Bruker APEXII CCD	Rigaku Oxford Diffraction Xcalibur, Sapphire3
Absorption correction	Multi-scan (*CrysAlis PRO*; Rigaku OD, 2018 ▸)	Multi-scan (*CrysAlis PRO*; Rigaku OD, 2018 ▸)
*T*_min_, *T*_max_	0.632, 1.000	0.980, 1.000
No. of measured, independent and observed [*I* > 2σ(*I*)] reflections	26211, 6560, 5274	27640, 4273, 1621
*R*_int_	0.025	0.070
(sin θ/λ)_max_ (Å^−1^)	0.707	0.595

Refinement		
*R*[*F* ^2^ > 2σ(*F* ^2^)], *wR*(*F* ^2^), *S*	0.046, 0.118, 1.03	0.047, 0.095, 1.04
No. of reflections	6560	4273
No. of parameters	297	307
No. of restraints	1	56
H-atom treatment	H atoms treated by a mixture of independent and constrained refinement	H atoms treated by a mixture of independent and constrained refinement
Δρ_max_, Δρ_min_ (e Å^−3^)	0.37, −0.36	0.26, −0.19

Computer programs: *APEX2* (Bruker, 2005 ▸), *CrysAlis PRO* (Rigaku OD, 2018 ▸), *SHELXT2014/5* (Sheldrick, 2015*a* ▸), *SHELXL2018/1* (Sheldrick, 2015*b* ▸), *SIR92* (Altomare *et al.*, 1994 ▸), *DIAMOND* (Brandenburg, 2007 ▸), *Mercury* (Macrae *et al.*, 2006 ▸), *WinGX* (Farrugia, 2012 ▸) and *CrystalExplorer* (Spackman & Jayatilaka, 2009 ▸).

## supplementary crystallographic information

### Tetra-n-butylammonium 2,6-dioxo-1,2,3,6-tetrahydropyrimidine-4-carboxylate monohydrate (100K) Crystal data

**Table:**

C_16_H_36_N^+^·C_5_H_3_N_2_O_4_^−^·H_2_O	*F*(000) = 912
*M**_r_* = 415.57	*D*_x_ = 1.150 Mg m^−^^3^
Monoclinic, *P*2_1_/*c*	Mo *K*α radiation, λ = 0.71073 Å
*a* = 10.0905 (5) Å	Cell parameters from 17418 reflections
*b* = 14.8664 (8) Å	θ = 2.0–30.2°
*c* = 16.1261 (9) Å	µ = 0.08 mm^−^^1^
β = 97.347 (5)°	*T* = 100 K
*V* = 2399.2 (2) Å^3^	Irregular, colourless
*Z* = 4	0.31 × 0.18 × 0.16 mm

### Tetra-n-butylammonium 2,6-dioxo-1,2,3,6-tetrahydropyrimidine-4-carboxylate monohydrate (100K) Data collection

**Table:**

Bruker APEXII CCD diffractometer	6560 independent reflections
Radiation source: fine-focus sealed X-ray tube	5274 reflections with *I* > 2σ(*I*)
Detector resolution: 7.9 pixels mm^-1^	*R*_int_ = 0.025
φ and ω scans	θ_max_ = 30.1°, θ_min_ = 1.9°
Absorption correction: multi-scan (CrysAlisPro; Rigaku OD, 2018)	*h* = −14→13
*T*_min_ = 0.632, *T*_max_ = 1.000	*k* = −19→20
26211 measured reflections	*l* = −19→22

### Tetra-n-butylammonium 2,6-dioxo-1,2,3,6-tetrahydropyrimidine-4-carboxylate monohydrate (100K) Refinement

**Table:**

Refinement on *F*^2^	Primary atom site location: iterative
Least-squares matrix: full	Secondary atom site location: difference Fourier map
*R*[*F*^2^ > 2σ(*F*^2^)] = 0.046	Hydrogen site location: mixed
*wR*(*F*^2^) = 0.118	H atoms treated by a mixture of independent and constrained refinement
*S* = 1.03	*w* = 1/[σ^2^(*F*_o_^2^) + (0.0533*P*)^2^ + 0.9257*P*] where *P* = (*F*_o_^2^ + 2*F*_c_^2^)/3
6560 reflections	(Δ/σ)_max_ < 0.001
297 parameters	Δρ_max_ = 0.37 e Å^−^^3^
1 restraint	Δρ_min_ = −0.36 e Å^−^^3^

### Tetra-n-butylammonium 2,6-dioxo-1,2,3,6-tetrahydropyrimidine-4-carboxylate monohydrate (100K) Special details

**Table:**

Geometry. All esds (except the esd in the dihedral angle between two l.s. planes) are estimated using the full covariance matrix. The cell esds are taken into account individually in the estimation of esds in distances, angles and torsion angles; correlations between esds in cell parameters are only used when they are defined by crystal symmetry. An approximate (isotropic) treatment of cell esds is used for estimating esds involving l.s. planes.

### Tetra-n-butylammonium 2,6-dioxo-1,2,3,6-tetrahydropyrimidine-4-carboxylate monohydrate (100K) Fractional atomic coordinates and isotropic or equivalent isotropic displacement parameters (Å^2^)

**Table:**

	*x*	*y*	*z*	*U*_iso_*/*U*_eq_	Occ. (<1)
N1	0.66470 (9)	0.57393 (6)	0.44839 (5)	0.01392 (17)	
H1	0.6305 (15)	0.5427 (10)	0.4853 (9)	0.027 (4)*	
C2	0.80132 (10)	0.58337 (7)	0.46003 (6)	0.0153 (2)	
O2	0.87264 (8)	0.55019 (6)	0.51914 (5)	0.02277 (18)	
N3	0.85324 (9)	0.63250 (7)	0.39951 (6)	0.01872 (19)	
H3	0.9431 (17)	0.6356 (11)	0.4049 (10)	0.033 (4)*	
C4	0.78097 (12)	0.67160 (9)	0.32996 (8)	0.0257 (3)	
O4	0.83964 (9)	0.70891 (9)	0.27739 (7)	0.0466 (3)	
C5	0.63733 (11)	0.66355 (9)	0.32642 (8)	0.0234 (2)	
H5	0.5824 (16)	0.6918 (11)	0.2842 (10)	0.032 (4)*	
C6	0.58455 (10)	0.61536 (7)	0.38472 (6)	0.01468 (19)	
C7	0.43431 (10)	0.60106 (7)	0.38434 (6)	0.0162 (2)	
O7	0.35897 (8)	0.65773 (6)	0.34528 (6)	0.02556 (19)	
O8	0.40227 (8)	0.53413 (6)	0.42361 (5)	0.02183 (18)	
N10	0.16527 (9)	0.64890 (6)	0.71583 (6)	0.01999 (19)	
C11	0.23130 (12)	0.65152 (8)	0.80568 (7)	0.0252 (2)	
H11A	0.162731	0.666953	0.842093	0.030*	
H11B	0.298703	0.700260	0.811208	0.030*	
C12	0.29905 (18)	0.56466 (11)	0.83718 (10)	0.0440 (4)	
H12A	0.361837	0.544834	0.798430	0.053*	
H12B	0.231249	0.516936	0.839666	0.053*	
C13	0.3754 (2)	0.58039 (15)	0.92478 (12)	0.0700 (7)	
H13A	0.420281	0.523517	0.943974	0.084*	0.698 (4)
H13B	0.446069	0.625580	0.919995	0.084*	0.698 (4)
H13C	0.312396	0.612618	0.956597	0.084*	0.302 (4)
H13D	0.388045	0.520048	0.950543	0.084*	0.302 (4)
C14A	0.2987 (3)	0.60960 (18)	0.98669 (13)	0.0476 (7)	0.698 (4)
H14A	0.224674	0.567621	0.989942	0.071*	0.698 (4)
H14B	0.263000	0.669712	0.972396	0.071*	0.698 (4)
H14C	0.355099	0.611802	1.040852	0.071*	0.698 (4)
C14B	0.4798 (5)	0.6179 (4)	0.9408 (3)	0.0421 (15)	0.302 (4)
H14D	0.471116	0.680281	0.921200	0.063*	0.302 (4)
H14E	0.547700	0.587096	0.912845	0.063*	0.302 (4)
H14F	0.506507	0.617144	1.001350	0.063*	0.302 (4)
C15	0.26768 (12)	0.62951 (9)	0.65638 (8)	0.0259 (3)	
H15A	0.297867	0.566407	0.664641	0.031*	
H15B	0.222976	0.634978	0.598272	0.031*	
C16	0.39030 (12)	0.69031 (10)	0.66631 (9)	0.0332 (3)	
H16A	0.361841	0.753793	0.669715	0.040*	
H16B	0.447696	0.675243	0.718934	0.040*	
C17	0.46970 (14)	0.67900 (10)	0.59284 (9)	0.0347 (3)	
H17A	0.492567	0.614683	0.587486	0.042*	
H17B	0.413228	0.697239	0.540801	0.042*	
C18	0.59751 (16)	0.73424 (14)	0.60233 (11)	0.0524 (5)	
H18A	0.656351	0.713928	0.651862	0.079*	
H18B	0.575709	0.797908	0.608666	0.079*	
H18C	0.642885	0.726506	0.552580	0.079*	
C19	0.10337 (11)	0.74153 (7)	0.69748 (7)	0.0201 (2)	
H19A	0.043909	0.754953	0.740200	0.024*	
H19B	0.175833	0.786861	0.703483	0.024*	
C20	0.02375 (13)	0.75215 (9)	0.61164 (8)	0.0276 (3)	
H20A	−0.056570	0.713362	0.607492	0.033*	
H20B	0.078800	0.732696	0.568292	0.033*	
C21	−0.01832 (18)	0.84942 (10)	0.59638 (9)	0.0409 (4)	
H21A	0.062117	0.888186	0.603271	0.049*	
H21B	−0.075909	0.867821	0.638714	0.049*	
C22	−0.0936 (2)	0.86374 (12)	0.50956 (11)	0.0526 (5)	
H22A	−0.175693	0.827766	0.503399	0.079*	
H22B	−0.037301	0.845143	0.467404	0.079*	
H22C	−0.116462	0.927527	0.501895	0.079*	
C23	0.05896 (12)	0.57558 (8)	0.70357 (7)	0.0238 (2)	
H23A	0.015447	0.577728	0.644991	0.029*	
H23B	0.103353	0.516338	0.712323	0.029*	
C24	−0.04814 (14)	0.58272 (9)	0.76131 (8)	0.0301 (3)	
H24A	−0.083940	0.644790	0.759398	0.036*	
H24B	−0.008146	0.570105	0.819443	0.036*	
C25	−0.16213 (16)	0.51665 (11)	0.73614 (8)	0.0388 (4)	
H25A	−0.200163	0.528091	0.677417	0.047*	
H25B	−0.126628	0.454496	0.739651	0.047*	
C26	−0.27213 (18)	0.52528 (14)	0.79220 (10)	0.0524 (5)	
H26A	−0.309482	0.586185	0.787442	0.079*	
H26B	−0.234829	0.513751	0.850363	0.079*	
H26C	−0.342804	0.481451	0.774853	0.079*	
O1W	0.13336 (8)	0.63464 (7)	0.43187 (6)	0.02528 (19)	
H1WA	0.193 (2)	0.6418 (13)	0.4002 (12)	0.048 (5)*	
H1WB	0.144 (2)	0.5788 (15)	0.4471 (13)	0.054 (6)*	

### Tetra-n-butylammonium 2,6-dioxo-1,2,3,6-tetrahydropyrimidine-4-carboxylate monohydrate (100K) Atomic displacement parameters (Å^2^)

**Table:**

	*U*^11^	*U*^22^	*U*^33^	*U*^12^	*U*^13^	*U*^23^
N1	0.0109 (4)	0.0163 (4)	0.0148 (4)	−0.0007 (3)	0.0026 (3)	0.0026 (3)
C2	0.0119 (4)	0.0161 (5)	0.0181 (5)	−0.0002 (4)	0.0026 (4)	−0.0011 (4)
O2	0.0132 (4)	0.0317 (4)	0.0226 (4)	−0.0008 (3)	−0.0007 (3)	0.0078 (3)
N3	0.0109 (4)	0.0228 (5)	0.0230 (5)	−0.0005 (3)	0.0039 (3)	0.0052 (4)
C4	0.0167 (5)	0.0318 (6)	0.0292 (6)	−0.0005 (5)	0.0052 (4)	0.0130 (5)
O4	0.0195 (4)	0.0752 (8)	0.0465 (6)	−0.0017 (5)	0.0091 (4)	0.0391 (6)
C5	0.0143 (5)	0.0304 (6)	0.0252 (5)	0.0010 (4)	0.0014 (4)	0.0120 (5)
C6	0.0118 (4)	0.0152 (4)	0.0170 (5)	0.0001 (4)	0.0020 (4)	0.0000 (4)
C7	0.0121 (4)	0.0204 (5)	0.0158 (5)	−0.0010 (4)	0.0012 (4)	0.0018 (4)
O7	0.0139 (4)	0.0272 (4)	0.0350 (5)	0.0016 (3)	0.0009 (3)	0.0129 (4)
O8	0.0145 (4)	0.0270 (4)	0.0232 (4)	−0.0043 (3)	−0.0004 (3)	0.0102 (3)
N10	0.0175 (4)	0.0187 (4)	0.0225 (5)	0.0035 (4)	−0.0024 (3)	−0.0062 (4)
C11	0.0240 (6)	0.0257 (6)	0.0235 (6)	0.0031 (5)	−0.0058 (4)	−0.0094 (5)
C12	0.0537 (9)	0.0381 (8)	0.0336 (7)	0.0191 (7)	−0.0205 (7)	−0.0099 (6)
C13	0.0857 (15)	0.0685 (13)	0.0436 (10)	0.0356 (12)	−0.0387 (10)	−0.0153 (9)
C14A	0.0655 (16)	0.0519 (14)	0.0238 (10)	−0.0146 (12)	−0.0007 (10)	0.0023 (9)
C14B	0.030 (2)	0.042 (3)	0.047 (3)	−0.014 (2)	−0.022 (2)	0.000 (2)
C15	0.0186 (5)	0.0293 (6)	0.0287 (6)	0.0079 (5)	−0.0013 (4)	−0.0149 (5)
C16	0.0189 (5)	0.0451 (8)	0.0359 (7)	0.0006 (5)	0.0048 (5)	−0.0230 (6)
C17	0.0265 (6)	0.0421 (8)	0.0358 (7)	0.0029 (6)	0.0054 (5)	−0.0189 (6)
C18	0.0301 (7)	0.0788 (13)	0.0516 (9)	−0.0103 (8)	0.0173 (7)	−0.0362 (9)
C19	0.0175 (5)	0.0173 (5)	0.0264 (5)	0.0034 (4)	0.0055 (4)	−0.0032 (4)
C20	0.0311 (6)	0.0244 (6)	0.0270 (6)	0.0065 (5)	0.0017 (5)	0.0020 (5)
C21	0.0595 (10)	0.0311 (7)	0.0326 (7)	0.0181 (7)	0.0072 (7)	0.0056 (6)
C22	0.0720 (12)	0.0442 (9)	0.0404 (9)	0.0195 (9)	0.0023 (8)	0.0180 (7)
C23	0.0269 (6)	0.0190 (5)	0.0231 (5)	−0.0018 (4)	−0.0062 (4)	−0.0029 (4)
C24	0.0316 (7)	0.0304 (6)	0.0269 (6)	−0.0105 (5)	−0.0009 (5)	−0.0013 (5)
C25	0.0432 (8)	0.0486 (9)	0.0223 (6)	−0.0243 (7)	−0.0044 (5)	0.0026 (6)
C26	0.0499 (9)	0.0774 (13)	0.0296 (7)	−0.0363 (9)	0.0038 (7)	−0.0009 (8)
O1W	0.0137 (4)	0.0286 (5)	0.0344 (5)	0.0012 (3)	0.0063 (3)	0.0043 (4)

### Tetra-n-butylammonium 2,6-dioxo-1,2,3,6-tetrahydropyrimidine-4-carboxylate monohydrate (100K) Geometric parameters (Å, º)

**Table:**

N1—C6	1.3693 (13)	C15—H15A	0.9900
N1—C2	1.3745 (13)	C15—H15B	0.9900
N1—H1	0.860 (16)	C16—C17	1.5220 (18)
C2—O2	1.2226 (13)	C16—H16A	0.9900
C2—N3	1.3751 (14)	C16—H16B	0.9900
N3—C4	1.3851 (15)	C17—C18	1.520 (2)
N3—H3	0.901 (17)	C17—H17A	0.9900
C4—O4	1.2268 (14)	C17—H17B	0.9900
C4—C5	1.4482 (16)	C18—H18A	0.9800
C5—C6	1.3449 (15)	C18—H18B	0.9800
C5—H5	0.922 (16)	C18—H18C	0.9800
C6—C7	1.5301 (14)	C19—C20	1.5173 (17)
C7—O8	1.2441 (13)	C19—H19A	0.9900
C7—O7	1.2498 (13)	C19—H19B	0.9900
N10—C11	1.5160 (15)	C20—C21	1.5182 (18)
N10—C15	1.5243 (15)	C20—H20A	0.9900
N10—C23	1.5243 (15)	C20—H20B	0.9900
N10—C19	1.5254 (14)	C21—C22	1.520 (2)
C11—C12	1.5177 (19)	C21—H21A	0.9900
C11—H11A	0.9900	C21—H21B	0.9900
C11—H11B	0.9900	C22—H22A	0.9800
C12—C13	1.538 (2)	C22—H22B	0.9800
C12—H12A	0.9900	C22—H22C	0.9800
C12—H12B	0.9900	C23—C24	1.5177 (19)
C13—C14B	1.191 (5)	C23—H23A	0.9900
C13—C14A	1.408 (3)	C23—H23B	0.9900
C13—H13A	0.9900	C24—C25	1.5274 (18)
C13—H13B	0.9900	C24—H24A	0.9900
C13—H13C	0.9900	C24—H24B	0.9900
C13—H13D	0.9900	C25—C26	1.524 (2)
C14A—H14A	0.9800	C25—H25A	0.9900
C14A—H14B	0.9800	C25—H25B	0.9900
C14A—H14C	0.9800	C26—H26A	0.9800
C14B—H14D	0.9800	C26—H26B	0.9800
C14B—H14E	0.9800	C26—H26C	0.9800
C14B—H14F	0.9800	O1W—H1WA	0.85 (2)
C15—C16	1.5242 (18)	O1W—H1WB	0.87 (2)
			
C6—N1—C2	122.84 (9)	H15A—C15—H15B	107.5
C6—N1—H1	120.7 (10)	C17—C16—C15	110.72 (11)
C2—N1—H1	116.4 (10)	C17—C16—H16A	109.5
O2—C2—N1	123.05 (9)	C15—C16—H16A	109.5
O2—C2—N3	121.87 (9)	C17—C16—H16B	109.5
N1—C2—N3	115.07 (9)	C15—C16—H16B	109.5
C2—N3—C4	126.07 (9)	H16A—C16—H16B	108.1
C2—N3—H3	115.3 (10)	C18—C17—C16	112.72 (11)
C4—N3—H3	118.5 (10)	C18—C17—H17A	109.0
O4—C4—N3	119.91 (11)	C16—C17—H17A	109.0
O4—C4—C5	125.39 (11)	C18—C17—H17B	109.0
N3—C4—C5	114.69 (10)	C16—C17—H17B	109.0
C6—C5—C4	120.02 (10)	H17A—C17—H17B	107.8
C6—C5—H5	120.3 (10)	C17—C18—H18A	109.5
C4—C5—H5	119.7 (10)	C17—C18—H18B	109.5
C5—C6—N1	121.00 (10)	H18A—C18—H18B	109.5
C5—C6—C7	123.50 (10)	C17—C18—H18C	109.5
N1—C6—C7	115.49 (9)	H18A—C18—H18C	109.5
O8—C7—O7	127.93 (10)	H18B—C18—H18C	109.5
O8—C7—C6	115.52 (9)	C20—C19—N10	115.27 (9)
O7—C7—C6	116.54 (9)	C20—C19—H19A	108.5
C11—N10—C15	110.83 (9)	N10—C19—H19A	108.5
C11—N10—C23	110.99 (9)	C20—C19—H19B	108.5
C15—N10—C23	107.80 (9)	N10—C19—H19B	108.5
C11—N10—C19	106.33 (8)	H19A—C19—H19B	107.5
C15—N10—C19	110.03 (9)	C19—C20—C21	110.58 (11)
C23—N10—C19	110.88 (9)	C19—C20—H20A	109.5
N10—C11—C12	114.97 (10)	C21—C20—H20A	109.5
N10—C11—H11A	108.5	C19—C20—H20B	109.5
C12—C11—H11A	108.5	C21—C20—H20B	109.5
N10—C11—H11B	108.5	H20A—C20—H20B	108.1
C12—C11—H11B	108.5	C20—C21—C22	112.28 (13)
H11A—C11—H11B	107.5	C20—C21—H21A	109.1
C11—C12—C13	109.23 (13)	C22—C21—H21A	109.1
C11—C12—H12A	109.8	C20—C21—H21B	109.1
C13—C12—H12A	109.8	C22—C21—H21B	109.1
C11—C12—H12B	109.8	H21A—C21—H21B	107.9
C13—C12—H12B	109.8	C21—C22—H22A	109.5
H12A—C12—H12B	108.3	C21—C22—H22B	109.5
C14B—C13—C12	126.5 (4)	H22A—C22—H22B	109.5
C14A—C13—C12	116.25 (19)	C21—C22—H22C	109.5
C14A—C13—H13A	108.2	H22A—C22—H22C	109.5
C12—C13—H13A	108.2	H22B—C22—H22C	109.5
C14A—C13—H13B	108.2	C24—C23—N10	114.52 (10)
C12—C13—H13B	108.2	C24—C23—H23A	108.6
H13A—C13—H13B	107.4	N10—C23—H23A	108.6
C14B—C13—H13C	105.7	C24—C23—H23B	108.6
C12—C13—H13C	105.7	N10—C23—H23B	108.6
C14B—C13—H13D	105.7	H23A—C23—H23B	107.6
C12—C13—H13D	105.7	C23—C24—C25	111.29 (11)
H13C—C13—H13D	106.1	C23—C24—H24A	109.4
C13—C14A—H14A	109.5	C25—C24—H24A	109.4
C13—C14A—H14B	109.5	C23—C24—H24B	109.4
H14A—C14A—H14B	109.5	C25—C24—H24B	109.4
C13—C14A—H14C	109.5	H24A—C24—H24B	108.0
H14A—C14A—H14C	109.5	C26—C25—C24	111.66 (13)
H14B—C14A—H14C	109.5	C26—C25—H25A	109.3
C13—C14B—H14D	109.5	C24—C25—H25A	109.3
C13—C14B—H14E	109.5	C26—C25—H25B	109.3
H14D—C14B—H14E	109.5	C24—C25—H25B	109.3
C13—C14B—H14F	109.5	H25A—C25—H25B	107.9
H14D—C14B—H14F	109.5	C25—C26—H26A	109.5
H14E—C14B—H14F	109.5	C25—C26—H26B	109.5
C16—C15—N10	115.36 (9)	H26A—C26—H26B	109.5
C16—C15—H15A	108.4	C25—C26—H26C	109.5
N10—C15—H15A	108.4	H26A—C26—H26C	109.5
C16—C15—H15B	108.4	H26B—C26—H26C	109.5
N10—C15—H15B	108.4	H1WA—O1W—H1WB	102.7 (18)
			
C6—N1—C2—O2	176.23 (10)	N10—C11—C12—C13	173.31 (15)
C6—N1—C2—N3	−4.41 (15)	C11—C12—C13—C14B	−76.8 (4)
O2—C2—N3—C4	179.14 (12)	C11—C12—C13—C14A	59.0 (3)
N1—C2—N3—C4	−0.22 (16)	C11—N10—C15—C16	−52.03 (14)
C2—N3—C4—O4	−175.20 (13)	C23—N10—C15—C16	−173.69 (11)
C2—N3—C4—C5	4.54 (18)	C19—N10—C15—C16	65.28 (13)
O4—C4—C5—C6	175.13 (14)	N10—C15—C16—C17	−167.64 (11)
N3—C4—C5—C6	−4.60 (19)	C15—C16—C17—C18	−176.63 (14)
C4—C5—C6—N1	0.54 (18)	C11—N10—C19—C20	−176.33 (10)
C4—C5—C6—C7	−178.16 (11)	C15—N10—C19—C20	63.60 (12)
C2—N1—C6—C5	4.30 (16)	C23—N10—C19—C20	−55.57 (13)
C2—N1—C6—C7	−176.90 (9)	N10—C19—C20—C21	−173.01 (11)
C5—C6—C7—O8	157.63 (11)	C19—C20—C21—C22	177.68 (13)
N1—C6—C7—O8	−21.14 (14)	C11—N10—C23—C24	55.88 (13)
C5—C6—C7—O7	−22.68 (16)	C15—N10—C23—C24	177.43 (10)
N1—C6—C7—O7	158.55 (10)	C19—N10—C23—C24	−62.08 (13)
C15—N10—C11—C12	−60.83 (15)	N10—C23—C24—C25	170.92 (11)
C23—N10—C11—C12	58.93 (15)	C23—C24—C25—C26	−178.31 (13)
C19—N10—C11—C12	179.62 (12)		

### Tetra-n-butylammonium 2,6-dioxo-1,2,3,6-tetrahydropyrimidine-4-carboxylate monohydrate (100K) Hydrogen-bond geometry (Å, º)

**Table:**

*D*—H···*A*	*D*—H	H···*A*	*D*···*A*	*D*—H···*A*
N1—H1···O8^i^	0.860 (16)	1.924 (16)	2.7668 (12)	166.4 (14)
N3—H3···O1*W*^ii^	0.901 (17)	1.913 (17)	2.8081 (12)	171.8 (15)
C11—H11*B*···O7^iii^	0.99	2.25	3.1462 (15)	151
C19—H19*A*···O4^iv^	0.99	2.28	3.1878 (14)	151
C23—H23*A*···O2^v^	0.99	2.37	3.3305 (14)	164
C24—H24*A*···O4^iv^	0.99	2.34	3.3197 (19)	171
O1*W*—H1*WA*···O7	0.85 (2)	2.00 (2)	2.8396 (12)	169.2 (18)
O1*W*—H1*WA*···O8	0.85 (2)	2.64 (2)	3.1155 (12)	117.3 (15)
O1*W*—H1*WB*···O2^i^	0.87 (2)	2.01 (2)	2.8618 (13)	168.3 (19)

Symmetry codes: (i) −*x*+1, −*y*+1, −*z*+1; (ii) *x*+1, *y*, *z*; (iii) *x*, −*y*+3/2, *z*+1/2; (iv) *x*−1, −*y*+3/2, *z*+1/2; (v) *x*−1, *y*, *z*.

### (295K) Crystal data

**Table:**

C_16_H_36_N^+^·C_5_H_3_N_2_O_4_^−^·H_2_O	*F*(000) = 912
*M**_r_* = 415.57	*D*_x_ = 1.105 Mg m^−^^3^
Monoclinic, *P*2_1_/*c*	Mo *K*α radiation, λ = 0.71073 Å
*a* = 10.1335 (5) Å	Cell parameters from 3106 reflections
*b* = 14.6690 (7) Å	θ = 3.0–21.2°
*c* = 16.9205 (8) Å	µ = 0.08 mm^−^^1^
β = 96.630 (4)°	*T* = 295 K
*V* = 2498.4 (2) Å^3^	Block, colourless
*Z* = 4	0.55 × 0.23 × 0.09 mm

### (295K) Data collection

**Table:**

Rigaku Oxford Diffraction Xcalibur, Sapphire3 diffractometer	4273 independent reflections
Radiation source: fine-focus sealed X-ray tube	1621 reflections with *I* > 2σ(*I*)
Detector resolution: 16.3990 pixels mm^-1^	*R*_int_ = 0.070
ω and φ scans	θ_max_ = 25.0°, θ_min_ = 2.8°
Absorption correction: multi-scan (CrysAlisPro; Rigaku OD, 2018)	*h* = −12→12
*T*_min_ = 0.980, *T*_max_ = 1.000	*k* = −16→16
27640 measured reflections	*l* = −20→20

### (295K) Refinement

**Table:**

Refinement on *F*^2^	Primary atom site location: structure-invariant direct methods
Least-squares matrix: full	Secondary atom site location: difference Fourier map
*R*[*F*^2^ > 2σ(*F*^2^)] = 0.047	Hydrogen site location: mixed
*wR*(*F*^2^) = 0.095	H atoms treated by a mixture of independent and constrained refinement
*S* = 1.04	*w* = 1/[σ^2^(*F*_o_^2^) + (0.030*P*)^2^] where *P* = (*F*_o_^2^ + 2*F*_c_^2^)/3
4273 reflections	(Δ/σ)_max_ = 0.001
307 parameters	Δρ_max_ = 0.26 e Å^−^^3^
56 restraints	Δρ_min_ = −0.19 e Å^−^^3^

### (295K) Special details

**Table:**

Geometry. All esds (except the esd in the dihedral angle between two l.s. planes) are estimated using the full covariance matrix. The cell esds are taken into account individually in the estimation of esds in distances, angles and torsion angles; correlations between esds in cell parameters are only used when they are defined by crystal symmetry. An approximate (isotropic) treatment of cell esds is used for estimating esds involving l.s. planes.
Refinement. Please see the Supporting Information for a full description of the refinement of the disorder assembly involving one of the n-butyl groups.

### (295K) Fractional atomic coordinates and isotropic or equivalent isotropic displacement parameters (Å^2^)

**Table:**

	*x*	*y*	*z*	*U*_iso_*/*U*_eq_	Occ. (<1)
N1	0.65997 (17)	0.57519 (13)	0.44599 (11)	0.0472 (5)	
H1	0.6254 (18)	0.5453 (13)	0.4826 (12)	0.057*	
C2	0.7952 (2)	0.58205 (16)	0.45510 (17)	0.0499 (6)	
O2	0.86644 (14)	0.55274 (11)	0.51216 (10)	0.0734 (6)	
N3	0.84553 (18)	0.62622 (13)	0.39389 (14)	0.0579 (6)	
H3	0.931 (2)	0.6305 (14)	0.3974 (12)	0.069*	
C4	0.7732 (3)	0.65988 (18)	0.32579 (19)	0.0774 (8)	
O4	0.83182 (17)	0.69171 (15)	0.27272 (13)	0.1322 (9)	
C5	0.6322 (2)	0.65389 (19)	0.32529 (17)	0.0718 (8)	
H5	0.578 (2)	0.6779 (14)	0.2804 (13)	0.086*	
C6	0.5795 (2)	0.61233 (15)	0.38384 (15)	0.0479 (6)	
C7	0.4310 (2)	0.60074 (19)	0.38728 (16)	0.0555 (7)	
O7	0.35729 (15)	0.65242 (12)	0.34479 (11)	0.0857 (6)	
O8	0.39840 (14)	0.54039 (12)	0.43105 (10)	0.0815 (6)	
N10	0.14286 (19)	0.65933 (14)	0.70428 (12)	0.0630 (6)	
C11	0.2037 (3)	0.66598 (19)	0.79054 (16)	0.0841 (9)	
H11A	0.266990	0.715812	0.794999	0.101*	
H11B	0.133751	0.681476	0.822808	0.101*	
C12	0.2727 (3)	0.5820 (2)	0.82472 (19)	0.1144 (11)	
H12A	0.207327	0.535090	0.831215	0.137*	0.55
H12B	0.331876	0.559208	0.788087	0.137*	0.55
H12C	0.355850	0.573372	0.802451	0.137*	0.45
H12D	0.217376	0.528770	0.812206	0.137*	0.45
C13A	0.3493 (13)	0.6010 (10)	0.9019 (6)	0.193 (4)	0.55
H13A	0.427192	0.635204	0.891178	0.232*	0.2
H13B	0.295887	0.641730	0.930300	0.232*	0.2
H13E	0.410871	0.550797	0.913505	0.232*	0.35
H13F	0.402359	0.654935	0.895443	0.232*	0.35
C14A	0.2872 (13)	0.6139 (10)	0.9661 (6)	0.180 (4)	0.35
H14A	0.351458	0.625437	1.011269	0.270*	0.35
H14B	0.228369	0.665130	0.957613	0.270*	0.35
H14C	0.236933	0.560366	0.975783	0.270*	0.35
C13B	0.2992 (16)	0.5948 (12)	0.9159 (7)	0.193 (4)	0.45
H13C	0.329722	0.656607	0.927175	0.232*	0.2
H13D	0.216411	0.586889	0.938731	0.232*	0.2
H13G	0.240945	0.640475	0.934596	0.232*	0.25
H13H	0.288846	0.537957	0.943909	0.232*	0.25
C14B	0.4340 (15)	0.6244 (12)	0.9250 (10)	0.182 (4)	0.25
H14D	0.462783	0.634885	0.980312	0.272*	0.25
H14E	0.488744	0.578362	0.905009	0.272*	0.25
H14F	0.441292	0.679919	0.895783	0.272*	0.25
C14C	0.3913 (10)	0.5353 (6)	0.9520 (6)	0.180 (4)	0.4
H14G	0.453563	0.497510	0.928456	0.270*	0.2
H14H	0.433849	0.561535	1.000342	0.270*	0.2
H14J	0.317066	0.499029	0.963613	0.270*	0.2
H14K	0.347631	0.480510	0.965991	0.270*	0.2
H14L	0.455048	0.520762	0.916123	0.270*	0.2
H14M	0.435875	0.562833	0.999198	0.270*	0.2
C15	0.2480 (2)	0.63645 (16)	0.65027 (15)	0.0750 (8)	
H15A	0.205410	0.632749	0.596007	0.090*	
H15B	0.283681	0.576591	0.664703	0.090*	
C16	0.3631 (3)	0.70328 (19)	0.65243 (17)	0.0967 (9)	
H16A	0.328748	0.762690	0.635352	0.116*	
H16B	0.404331	0.709057	0.706854	0.116*	
C17	0.4627 (3)	0.6762 (2)	0.6026 (2)	0.1374 (13)	
H17A	0.420044	0.669633	0.548602	0.165*	
H17B	0.495856	0.616595	0.620048	0.165*	
C18	0.5782 (3)	0.7383 (2)	0.60122 (19)	0.1481 (14)	
H18A	0.635629	0.732614	0.650213	0.178*	
H18B	0.547327	0.800037	0.595035	0.178*	
H18C	0.626182	0.722348	0.557510	0.178*	
C19	0.0819 (2)	0.75089 (16)	0.68169 (16)	0.0702 (8)	
H19A	0.018345	0.765289	0.718533	0.084*	
H19B	0.151486	0.796579	0.688643	0.084*	
C20	0.0128 (3)	0.75878 (18)	0.59841 (18)	0.0884 (9)	
H20A	0.073923	0.741475	0.560917	0.106*	
H20B	−0.061720	0.716915	0.591922	0.106*	
C21	−0.0359 (3)	0.8531 (2)	0.58024 (19)	0.1206 (12)	
H21A	0.038787	0.894827	0.587842	0.145*	
H21B	−0.097424	0.869888	0.617650	0.145*	
C22	−0.1041 (4)	0.8642 (2)	0.4970 (2)	0.1652 (16)	
H22A	−0.184583	0.829057	0.490978	0.198*	
H22B	−0.046435	0.843303	0.459575	0.198*	
H22C	−0.124951	0.927360	0.487189	0.198*	
C23	0.0391 (2)	0.58363 (15)	0.69378 (15)	0.0750 (8)	
H23A	0.083208	0.525582	0.704889	0.090*	
H23B	0.000089	0.582772	0.638666	0.090*	
C24	−0.0726 (3)	0.5930 (2)	0.7470 (2)	0.1180 (12)	
H24A	−0.033422	0.594611	0.802102	0.142*	
H24B	−0.117241	0.650779	0.735494	0.142*	
C25	−0.1686 (3)	0.5225 (2)	0.7383 (2)	0.1292 (12)	
H25A	−0.123636	0.464660	0.749059	0.155*	
H25B	−0.208397	0.521395	0.683314	0.155*	
C26	−0.2779 (3)	0.5302 (2)	0.79096 (19)	0.1455 (14)	
H26A	−0.317042	0.589774	0.785299	0.175*	
H26B	−0.241944	0.520775	0.845354	0.175*	
H26C	−0.344529	0.484942	0.775853	0.175*	
O1W	0.12369 (17)	0.63080 (13)	0.42446 (14)	0.0860 (7)	
H1WA	0.188 (2)	0.6385 (17)	0.3966 (15)	0.103*	
H1WB	0.139 (3)	0.5857 (19)	0.4461 (17)	0.103*	

### (295K) Atomic displacement parameters (Å^2^)

**Table:**

	*U*^11^	*U*^22^	*U*^33^	*U*^12^	*U*^13^	*U*^23^
N1	0.0313 (11)	0.0604 (15)	0.0514 (15)	−0.0036 (10)	0.0107 (10)	0.0089 (11)
C2	0.0380 (17)	0.0521 (18)	0.062 (2)	−0.0062 (13)	0.0139 (14)	−0.0010 (15)
O2	0.0381 (9)	0.1015 (14)	0.0779 (13)	−0.0049 (9)	−0.0047 (9)	0.0245 (11)
N3	0.0333 (11)	0.0624 (14)	0.0797 (16)	−0.0024 (11)	0.0139 (13)	0.0126 (13)
C4	0.054 (2)	0.088 (2)	0.093 (2)	−0.0012 (16)	0.0212 (18)	0.0369 (19)
O4	0.0754 (14)	0.197 (2)	0.131 (2)	−0.0048 (14)	0.0396 (13)	0.0974 (18)
C5	0.0474 (18)	0.096 (2)	0.073 (2)	−0.0019 (14)	0.0113 (14)	0.0428 (18)
C6	0.0376 (14)	0.0489 (17)	0.0566 (17)	0.0033 (12)	0.0027 (13)	0.0102 (14)
C7	0.0401 (16)	0.067 (2)	0.0598 (19)	−0.0025 (14)	0.0059 (13)	0.0134 (15)
O7	0.0449 (10)	0.0949 (14)	0.1154 (16)	0.0094 (9)	0.0011 (10)	0.0499 (12)
O8	0.0428 (10)	0.1129 (15)	0.0883 (14)	−0.0103 (10)	0.0052 (9)	0.0527 (12)
N10	0.0656 (13)	0.0544 (15)	0.0706 (16)	0.0000 (12)	0.0148 (12)	−0.0165 (12)
C11	0.095 (2)	0.091 (2)	0.066 (2)	−0.0159 (18)	0.0094 (17)	−0.0229 (18)
C12	0.125 (3)	0.107 (3)	0.102 (3)	−0.001 (2)	−0.030 (2)	0.001 (2)
C13A	0.217 (10)	0.237 (6)	0.106 (5)	−0.034 (6)	−0.061 (6)	0.041 (5)
C14A	0.222 (9)	0.196 (9)	0.108 (5)	−0.028 (6)	−0.046 (6)	0.025 (6)
C13B	0.218 (10)	0.237 (6)	0.106 (5)	−0.035 (6)	−0.060 (6)	0.042 (5)
C14B	0.221 (9)	0.197 (9)	0.109 (6)	−0.029 (6)	−0.052 (6)	0.025 (6)
C14C	0.220 (9)	0.194 (9)	0.110 (5)	−0.026 (6)	−0.048 (6)	0.032 (6)
C15	0.0656 (18)	0.078 (2)	0.082 (2)	0.0122 (15)	0.0130 (15)	−0.0291 (16)
C16	0.078 (2)	0.116 (3)	0.102 (2)	−0.0080 (19)	0.0348 (18)	−0.030 (2)
C17	0.089 (2)	0.171 (4)	0.161 (3)	−0.008 (2)	0.049 (2)	−0.056 (3)
C18	0.093 (2)	0.212 (4)	0.149 (3)	−0.030 (3)	0.054 (2)	−0.055 (3)
C19	0.0719 (17)	0.0452 (19)	0.098 (2)	0.0068 (14)	0.0280 (16)	−0.0108 (17)
C20	0.087 (2)	0.070 (2)	0.109 (3)	0.0185 (17)	0.0141 (18)	0.0040 (19)
C21	0.155 (3)	0.092 (3)	0.117 (3)	0.033 (2)	0.021 (2)	0.016 (2)
C22	0.200 (4)	0.135 (3)	0.158 (4)	0.048 (3)	0.007 (3)	0.046 (3)
C23	0.0700 (18)	0.0534 (18)	0.099 (2)	−0.0105 (15)	0.0007 (16)	−0.0140 (16)
C24	0.086 (2)	0.098 (2)	0.177 (4)	−0.023 (2)	0.043 (2)	−0.036 (2)
C25	0.117 (3)	0.123 (3)	0.150 (3)	−0.039 (2)	0.028 (2)	−0.002 (2)
C26	0.115 (3)	0.202 (4)	0.125 (3)	−0.057 (3)	0.039 (2)	−0.007 (3)
O1W	0.0474 (11)	0.0889 (17)	0.125 (2)	0.0066 (11)	0.0233 (10)	0.0163 (14)

### (295K) Geometric parameters (Å, º)

**Table:**

N1—C2	1.365 (2)	C14C—H14G	0.9600
N1—C6	1.367 (2)	C14C—H14H	0.9600
N1—H1	0.866 (19)	C14C—H14J	0.9600
C2—O2	1.215 (2)	C14C—H14K	0.9600
C2—N3	1.369 (3)	C14C—H14L	0.9600
N3—C4	1.384 (3)	C14C—H14M	0.9600
N3—H3	0.86 (2)	C15—C16	1.520 (3)
C4—O4	1.225 (3)	C15—H15A	0.9700
C4—C5	1.430 (3)	C15—H15B	0.9700
C5—C6	1.327 (3)	C16—C17	1.443 (3)
C5—H5	0.95 (2)	C16—H16A	0.9700
C6—C7	1.523 (3)	C16—H16B	0.9700
C7—O8	1.224 (2)	C17—C18	1.485 (3)
C7—O7	1.235 (2)	C17—H17A	0.9700
N10—C19	1.509 (3)	C17—H17B	0.9700
N10—C15	1.519 (3)	C18—H18A	0.9600
N10—C11	1.520 (3)	C18—H18B	0.9600
N10—C23	1.526 (2)	C18—H18C	0.9600
C11—C12	1.500 (3)	C19—C20	1.504 (3)
C11—H11A	0.9700	C19—H19A	0.9700
C11—H11B	0.9700	C19—H19B	0.9700
C12—C13A	1.468 (9)	C20—C21	1.490 (3)
C12—C13B	1.547 (11)	C20—H20A	0.9700
C12—H12A	0.9700	C20—H20B	0.9700
C12—H12B	0.9700	C21—C22	1.505 (4)
C12—H12C	0.9700	C21—H21A	0.9700
C12—H12D	0.9700	C21—H21B	0.9700
C13A—C14C	1.321 (12)	C22—H22A	0.9600
C13A—C14A	1.330 (13)	C22—H22B	0.9600
C13A—H13A	0.9700	C22—H22C	0.9600
C13A—H13B	0.9700	C23—C24	1.532 (3)
C13A—H13E	0.9700	C23—H23A	0.9700
C13A—H13F	0.9700	C23—H23B	0.9700
C14A—H14A	0.9600	C24—C25	1.416 (3)
C14A—H14B	0.9600	C24—H24A	0.9700
C14A—H14C	0.9600	C24—H24B	0.9700
C13B—C14C	1.369 (13)	C25—C26	1.504 (4)
C13B—C14B	1.425 (15)	C25—H25A	0.9700
C13B—H13C	0.9700	C25—H25B	0.9700
C13B—H13D	0.9700	C26—H26A	0.9600
C13B—H13G	0.9700	C26—H26B	0.9600
C13B—H13H	0.9700	C26—H26C	0.9600
C14B—H14D	0.9600	O1W—H1WA	0.85 (2)
C14B—H14E	0.9600	O1W—H1WB	0.76 (3)
C14B—H14F	0.9600		
			
C2—N1—C6	124.0 (2)	H14G—C14C—H14H	109.5
C2—N1—H1	116.2 (13)	C13A—C14C—H14J	109.5
C6—N1—H1	119.8 (13)	H14G—C14C—H14J	109.5
O2—C2—N1	124.1 (2)	H14H—C14C—H14J	109.5
O2—C2—N3	122.0 (2)	C13B—C14C—H14K	109.5
N1—C2—N3	114.0 (2)	C13B—C14C—H14L	109.5
C2—N3—C4	126.2 (2)	H14K—C14C—H14L	109.5
C2—N3—H3	116.1 (14)	C13B—C14C—H14M	109.5
C4—N3—H3	117.6 (15)	H14K—C14C—H14M	109.5
O4—C4—N3	119.4 (2)	H14L—C14C—H14M	109.5
O4—C4—C5	126.0 (3)	N10—C15—C16	115.61 (19)
N3—C4—C5	114.6 (3)	N10—C15—H15A	108.4
C6—C5—C4	120.8 (2)	C16—C15—H15A	108.4
C6—C5—H5	121.5 (13)	N10—C15—H15B	108.4
C4—C5—H5	117.7 (13)	C16—C15—H15B	108.4
C5—C6—N1	120.1 (2)	H15A—C15—H15B	107.4
C5—C6—C7	124.4 (2)	C17—C16—C15	113.2 (2)
N1—C6—C7	115.5 (2)	C17—C16—H16A	108.9
O8—C7—O7	127.5 (2)	C15—C16—H16A	108.9
O8—C7—C6	116.2 (2)	C17—C16—H16B	108.9
O7—C7—C6	116.3 (2)	C15—C16—H16B	108.9
C19—N10—C15	109.83 (19)	H16A—C16—H16B	107.7
C19—N10—C11	107.1 (2)	C16—C17—C18	116.5 (3)
C15—N10—C11	110.92 (18)	C16—C17—H17A	108.2
C19—N10—C23	111.21 (17)	C18—C17—H17A	108.2
C15—N10—C23	106.93 (18)	C16—C17—H17B	108.2
C11—N10—C23	110.85 (19)	C18—C17—H17B	108.2
C12—C11—N10	115.9 (2)	H17A—C17—H17B	107.3
C12—C11—H11A	108.3	C17—C18—H18A	109.5
N10—C11—H11A	108.3	C17—C18—H18B	109.5
C12—C11—H11B	108.3	H18A—C18—H18B	109.5
N10—C11—H11B	108.3	C17—C18—H18C	109.5
H11A—C11—H11B	107.4	H18A—C18—H18C	109.5
C13A—C12—C11	111.3 (6)	H18B—C18—H18C	109.5
C11—C12—C13B	107.7 (6)	C20—C19—N10	116.0 (2)
C13A—C12—H12A	109.4	C20—C19—H19A	108.3
C11—C12—H12A	109.4	N10—C19—H19A	108.3
C13A—C12—H12B	109.4	C20—C19—H19B	108.3
C11—C12—H12B	109.4	N10—C19—H19B	108.3
H12A—C12—H12B	108.0	H19A—C19—H19B	107.4
C11—C12—H12C	110.2	C21—C20—C19	111.9 (2)
C13B—C12—H12C	110.2	C21—C20—H20A	109.2
C11—C12—H12D	110.2	C19—C20—H20A	109.2
C13B—C12—H12D	110.2	C21—C20—H20B	109.2
H12C—C12—H12D	108.5	C19—C20—H20B	109.2
C14C—C13A—C12	122.0 (12)	H20A—C20—H20B	107.9
C14A—C13A—C12	120.2 (12)	C20—C21—C22	113.5 (3)
C14C—C13A—H13A	106.8	C20—C21—H21A	108.9
C12—C13A—H13A	106.8	C22—C21—H21A	108.9
C14C—C13A—H13B	106.8	C20—C21—H21B	108.9
C12—C13A—H13B	106.8	C22—C21—H21B	108.9
H13A—C13A—H13B	106.7	H21A—C21—H21B	107.7
C14A—C13A—H13E	107.3	C21—C22—H22A	109.5
C12—C13A—H13E	107.3	C21—C22—H22B	109.5
C14A—C13A—H13F	107.3	H22A—C22—H22B	109.5
C12—C13A—H13F	107.3	C21—C22—H22C	109.5
H13E—C13A—H13F	106.9	H22A—C22—H22C	109.5
C13A—C14A—H14A	109.5	H22B—C22—H22C	109.5
C13A—C14A—H14B	109.5	N10—C23—C24	114.4 (2)
H14A—C14A—H14B	109.5	N10—C23—H23A	108.7
C13A—C14A—H14C	109.5	C24—C23—H23A	108.7
H14A—C14A—H14C	109.5	N10—C23—H23B	108.7
H14B—C14A—H14C	109.5	C24—C23—H23B	108.7
C14C—C13B—C12	113.5 (12)	H23A—C23—H23B	107.6
C14B—C13B—C12	101.4 (12)	C25—C24—C23	114.7 (3)
C14C—C13B—H13C	108.9	C25—C24—H24A	108.6
C12—C13B—H13C	108.9	C23—C24—H24A	108.6
C14C—C13B—H13D	108.9	C25—C24—H24B	108.6
C12—C13B—H13D	108.9	C23—C24—H24B	108.6
H13C—C13B—H13D	107.7	H24A—C24—H24B	107.6
C14B—C13B—H13G	111.5	C24—C25—C26	115.3 (3)
C12—C13B—H13G	111.5	C24—C25—H25A	108.5
C14B—C13B—H13H	111.5	C26—C25—H25A	108.5
C12—C13B—H13H	111.5	C24—C25—H25B	108.5
H13G—C13B—H13H	109.3	C26—C25—H25B	108.5
C13B—C14B—H14D	109.5	H25A—C25—H25B	107.5
C13B—C14B—H14E	109.5	C25—C26—H26A	109.5
H14D—C14B—H14E	109.5	C25—C26—H26B	109.5
C13B—C14B—H14F	109.5	H26A—C26—H26B	109.5
H14D—C14B—H14F	109.5	C25—C26—H26C	109.5
H14E—C14B—H14F	109.5	H26A—C26—H26C	109.5
C13A—C14C—H14G	109.5	H26B—C26—H26C	109.5
C13A—C14C—H14H	109.5	H1WA—O1W—H1WB	105 (3)
			
C6—N1—C2—O2	176.4 (2)	N10—C11—C12—C13B	−167.6 (7)
C6—N1—C2—N3	−3.0 (3)	C11—C12—C13A—C14C	163.1 (11)
O2—C2—N3—C4	178.3 (2)	C11—C12—C13A—C14A	73.6 (15)
N1—C2—N3—C4	−2.2 (3)	C11—C12—C13B—C14C	−163.6 (11)
C2—N3—C4—O4	−174.3 (3)	C11—C12—C13B—C14B	−98.0 (12)
C2—N3—C4—C5	6.1 (4)	C19—N10—C15—C16	60.0 (3)
O4—C4—C5—C6	175.4 (3)	C11—N10—C15—C16	−58.2 (3)
N3—C4—C5—C6	−5.0 (4)	C23—N10—C15—C16	−179.2 (2)
C4—C5—C6—N1	0.5 (4)	N10—C15—C16—C17	177.5 (2)
C4—C5—C6—C7	−178.5 (2)	C15—C16—C17—C18	179.7 (3)
C2—N1—C6—C5	3.9 (3)	C15—N10—C19—C20	60.8 (3)
C2—N1—C6—C7	−177.1 (2)	C11—N10—C19—C20	−178.6 (2)
C5—C6—C7—O8	159.8 (3)	C23—N10—C19—C20	−57.4 (3)
N1—C6—C7—O8	−19.2 (3)	N10—C19—C20—C21	−176.2 (2)
C5—C6—C7—O7	−19.2 (4)	C19—C20—C21—C22	179.2 (3)
N1—C6—C7—O7	161.7 (2)	C19—N10—C23—C24	−62.8 (3)
C19—N10—C11—C12	−178.8 (2)	C15—N10—C23—C24	177.3 (2)
C15—N10—C11—C12	−58.9 (3)	C11—N10—C23—C24	56.3 (3)
C23—N10—C11—C12	59.7 (3)	N10—C23—C24—C25	−179.4 (3)
N10—C11—C12—C13A	168.9 (7)	C23—C24—C25—C26	179.3 (3)

### (295K) Hydrogen-bond geometry (Å, º)

**Table:**

*D*—H···*A*	*D*—H	H···*A*	*D*···*A*	*D*—H···*A*
N1—H1···O8^i^	0.866 (19)	1.96 (2)	2.800 (3)	162.1 (18)
N3—H3···O1*W*^ii^	0.86 (2)	1.96 (2)	2.807 (2)	170 (2)
C11—H11*A*···O7^iii^	0.97	2.26	3.168 (3)	155
C19—H19*A*···O4^iv^	0.97	2.28	3.226 (3)	164
C23—H23*B*···O2^v^	0.97	2.44	3.386 (3)	166
C24—H24*B*···O4^iv^	0.97	2.47	3.346 (4)	151
O1*W*—H1*WA*···O7	0.85 (2)	2.03 (2)	2.874 (2)	172 (2)
O1*W*—H1*WA*···O8	0.85 (2)	2.59 (3)	3.074 (2)	118 (2)
O1*W*—H1*WB*···O2^i^	0.76 (3)	2.15 (3)	2.895 (2)	164 (3)

Symmetry codes: (i) −*x*+1, −*y*+1, −*z*+1; (ii) *x*+1, *y*, *z*; (iii) *x*, −*y*+3/2, *z*+1/2; (iv) *x*−1, −*y*+3/2, *z*+1/2; (v) *x*−1, *y*, *z*.
