# Supplementary material for: Tetra-n-butyl­ammonium orotate monohydrate: knowledge-based comparison of the results of accurate and lower-resolution analyses and a non-routine disorder refinement
Source: Acta Crystallogr E Crystallogr Commun. 2019 Oct 8;75(Pt 11):1632–7. doi: 10.1107/S2056989019013380 (PMC6829738; doi:10.1107/S2056989019013380)
Supplement: Supplementary file 5 [file e-75-01632-sup5.pdf]

Tetra-*n*-butylammonium orotate hydrate: knowledge-based comparison of the results of accurate and lower-resolution analyses and a non-routine disorder refinement

Irene Ara,[a] Zeineb Basdouri,[b,c] Larry R. Falvello,[c] Mohsen Graia,[b,d] Pablo Guerra[c] and Milagros Tomás[a]

[a] Instituto de Síntesis Química y Catálisis Homogénea (ISQCH), C.S.I.C.-University of Zaragoza, Departamento de Química Inorgánica, Pedro Cerbuna 12, E-50009, Zaragoza, Spain.
[b] Laboratoire de matériaux, Cristallochimie et thermodynamique appliquée, Département de chimie, Faculté des Sciences de Tunis, Université de Tunis El Manar, 2092 El Manar II Tunis, Tunisia.
[c] University of Zaragoza -C.S.I.C., Instituto de Ciencia de Materiales de Aragón (ICMA), Departamento de Química Inorgánica, E-50009 Zaragoza, Spain.
[d] Université de Sfax, Faculté de Sciences de Sfax, Route de la Soukra Km 4, Sfax 3038, Tunisia.

SUPPORTING INFORMATION

CONTENTS

I. Refinement of the disordered terminal ethyl fragment in the $^{n}Bu_4N^+$ cation at T = 295 K, 2.

II. Results of Mogul geometry check for ($^{n}Bu_4N$)(Horot)·H_2O at T = 100 K, 1 (internal code ial12p-1).

III. Results of Mogul geometry check for ($^{n}Bu_4N$)(Horot)·H_2O at T = 295 K, 2 (internal code est4l).

Refinement of the disordered terminal ethyl fragment in the $^{n}Bu_4N^+$ cation at T = 295 K, 2.

For the structure of ($^{n}Bu_4N$)(Horot)·H_2O at T = 100 K, **1**, the cation presents a simple two-way disorder of the terminal methyl group at C14, Fig. S1. This type of disorder is common and is handled routinely by refining the populations of the two disorder groups such that they sum to 1.0. The two disordered congeners of C14 were observed in a difference map. The H atoms of the neighboring CH_2 group at C13 were placed at calculated positions such that one pair of H atoms corresponds to the geometry required by the principal component of the disordered methyl group (pink in Fig. S1), and the other corresponds to the minor component (C14B, light blue in Fig. S1). The populations of the two pairs of CH_2 H atoms were tied to those of their respective methyl-group neighbors. The refined value of the population of the major component, C14A, was 0.698 (4).

It can be seen that the displacement ellipsoids in the C11 - C14 chain become more prolate -- more extended in a transverse direction -- toward the periphery of the chain. It is possible to split C13 into two components, but the resulting geometry is no more favorable than that involving the mean position used in our refinement. Another feature of the result is relatively common, if unwelcome -- the apparent bond distances C13---C14A, 1.408 (3) Å, and C13---C14B, 1.191 (5) Å, are shorter than expected, with both affected in the well-known way by both libration and the disorder.

At T = 295 K, the disorder is further developed, with C13 now sufficiently disordered to be represented naturally by two atomic sites -- observed sites, not calculated by splitting an atom. The more challenging aspect of the disorder at this temperature, however, arises with C14, which is now split into three atomic sites. Fig. S2 is a composite image of the entire disorder assembly. Here again, all non-hydrogen atomic sites were observed in a difference map.

The first task to be undertaken in such a case is to factor the overall set of atomic sites (the "disorder assembly" as per the Core CIF Dictionary) into chemically sound fragments ("disorder groups" in the Core CIF Dictionary) which populate this region of the structure but which are not simultaneously present in a given asymmetric unit. In this case, it was possible to identify four credible disorder groups:
1) C11/C12/C13A/C14A (pink in Fig. S3);
2) C11/C12/C13B/C14B (light blue in Fig. S3);
3) C11/C12/C13A/C14C (pink in Fig. S4);
4) C11/C12/C13B/C14C (light blue in Fig. S4).
The atoms of just one disorder group simultaneously populate a given asymmetric unit.

The same two principal factors must be addressed in a case such as this, as in a simpler case such as in the structure of **1**: a) Populations must be assigned to all of the atomic sites in such a way that the overall stoichiometry is correct for the known chemical fragment that comprises the disorder assembly and is correct also for each of the individual disorder groups. b) Those hydrogen atoms whose positions can be calculated reliably on the basis of the locations of the heavier atoms, should be thus placed. In the

present case this is complicated by the fact that the two congeners of C13 are bonded not only to methyl groups that can be identified individually with each of them -- namely C14A bonded to C13A and C14B to C13B -- but both are also bonded to a third methyl site common to both of them, namely C14C.

Our approach to the first item -- assigning populations to the disordered atomic sites, relied in the first instance on constrained refinement of populations. For example, the occupancies of C13A and C13B must sum to 1.0, as must the occupancies of C14A, C14B and C14C. We carried out a refinement dividing the atoms involved into the four groups defined above, refining their populations but with a strong restraint to a total population of 1.0. The final assignment of site occupancies was based on the result of this refinement; the only arbitrary assignment involved the common methyl group C14C, whose occupancy was divided equally between the two groups to which it belongs. The final occupancies of the four groups defined above are 1) 0.35; 2) 0.25; 3) 0.20; 4) 0.20. Atomic site occupancy factors were set on this basis, keeping in mind that some of the atoms belong to more than one disorder group. So for example, the occupancy of C13A was fixed at 0.55, the total for the two disorder groups, 1) and 3) above, to which it belongs.

As for the calculation of H-atom positions, it is necessary to invoke some program-specific terminology in order to describe how this was done. The refinement was conducted with ShelxL2018/1 (Sheldrick, 2015), which has a facility for assigning atoms to different "parts" whose geometries are calculated independently of each other. In a case of simple disorder such as that found for the structure at T = 100 K, **1**, a simple division of the disordered methyl groups and the correspondingly affected H atoms bonded to C13 into "PART 1" and "PART 2" permits the calculation of the H-atom positions, for which C13 must initially be bonded to exactly two other atoms. So when the positions of the pink H atoms are being calculated, Fig. S3, the program only considers C13 to be bonded to C12 and C14A.

For the more complex disorder at T = 295 K, **2**, the situation is more involved. One can assign C13A to "PART 1" and C13B to "PART 2" (Fig. S3, pink and blue, respectively); but C14C is also bonded to C13A and C13B, so each of them has bonds to three neighbors rather than the two required to permit the calculation of positions for two methylene H atoms on each of C13A and C13B.

A simple construct permits the calculation of all of the affected H atoms in the disorder assembly. There are five carbon atom sites, methylene C13A and C13B and methyl C14A, C14B and C14C. These were all assigned to different part numbers, namely C13A to PART 1, C13B to PART 2, C14A to PART 4, C14B to PART 5 and C14C to PART 3. In this way it is possible to exert individual control over which C---C bonds are considered relevant when generating H-atom positions, by using the instruction "BIND m n" which permits atoms from PART m to be considered bonded to atoms from PART n. The details can be found by examining the embedded instruction file within the CIF. As an example, C13A, in PART 1, needs two H atoms whose geometry is consistent with the presence of a methyl group at C14A (pink in Fig. S3). An AFIX instruction can be inserted with the two H atoms belonging to PART 4, with a "BIND 1 4" instruction having been included earlier in the input. The other atoms bonded to C13A do not interfere with the generation

of the two H atoms. Similarly, C13A needs two adjoined H atoms consistent with the presence of a methyl group at C14C. With a "BIND 1 3" instruction having been introduced earlier, C13A can be followed by an additional "AFIX 23" instruction with the two H atoms included in PART 3 (pink in Fig. S4).

Other approaches to modelling this disorder assembly will also work. The conceptually simple model described here was intended to model the average electron density in this region of the structure while still employing an atomic model. If it had become more complex, then other solutions, possibly including a non-atomic model (*i.e.*, Squeeze; Spek, 2015) could have been considered. In the actual event, while the overall geometrical results are typical of refinements of *n*-butyl groups, all of the C---C bond distances involving methyl groups are observed with the foreshortened distances [in the range 1.321 (12) - 1.425 (15) Å] commonly associated with disorder and/or libration. These "apparent" distances (a term that is invoked for disorder and especially for dynamic processes, Stebler & Bürgi, 1987) cannot be used as representative distances for C---C single bonds.

REFERENCES

Core CIF dictionary: https://www.iucr.org/resources/cif/dictionaries/cif_core
Definition of _atom_site_disorder_assembly:
https://www.iucr.org/__data/iucr/cifdic_html/1/cif_core.dic/Iatom_site_disorder_assembly.html
Definition of _atom_site_disorder_group:
https://www.iucr.org/__data/iucr/cifdic_html/1/cif_core.dic/Iatom_site_disorder_group.html

Sheldrick, G. M. (2015). *Acta Cryst.* C**71**, 3-8. doi: 10.1107/S2053229614024218

Spek, A. L. (2015). *Acta Cryst.* C**71**, 9-18. doi: 10.1107/S2053229614024929

Stebler, M. & Bürgi, H. B. (1987). *J. Am. Chem. Soc.* **109**, 1395-1401. doi: 10.1021/ja00239a020

FIGURE CAPTIONS

Figure S1. $^{n}Bu_4N^+$ cation from the structure of ($^{n}Bu_4N$)(Horot)·H_2O at T = 100 K, **1**. The disorder component shown in pink has a fractional population of 0.698 (4). Non-hydrogen atoms are represented by their 50% probability ellipsoids.

Figure S2. Composite image of the $^{n}Bu_4N^+$ cation from the structure of ($^{n}Bu_4N$)(Horot)·H_2O at T = 295 K, **2**, showing the positions of all of the atoms in the disorder assembly at the distal end of the C11 - C14 *n*-butyl group. Non-hydrogen atoms are represented by their 50% probability ellipsoids.

Figure S3. Composite image of the disorder assembly at C12/C13/C14 in the structure of (^{n}Bu$_4$N)(Horot)·H_2O at T = 295 K, **2**. Two of the disorder groups are identified by color, namely C12/C13A/C14A in pink and C12/C13B/C14B in light blue. Other than C12, which is represented by its 50% ellipsoid, the C atoms are shown as circles of arbitrary radius. H atoms are represented by smaller circles. Atoms and bonds not belonging to the fragments represented in pink and light blue, are shown in gray.

Figure S4. Composite image of the disorder assembly at C12/C13/C14 in the structure of (^{n}Bu$_4$N)(Horot)·H_2O at T = 295 K, **2**, now with the remaining two disorder groups identified by color. C12/C13A/C14C is drawn mostly in pink (C12 in gray), and C12/C13BC14C mostly in light blue. C14C belongs to both disorder groups and is drawn in pink with blue divisions.

FIGURES

Figure S1.

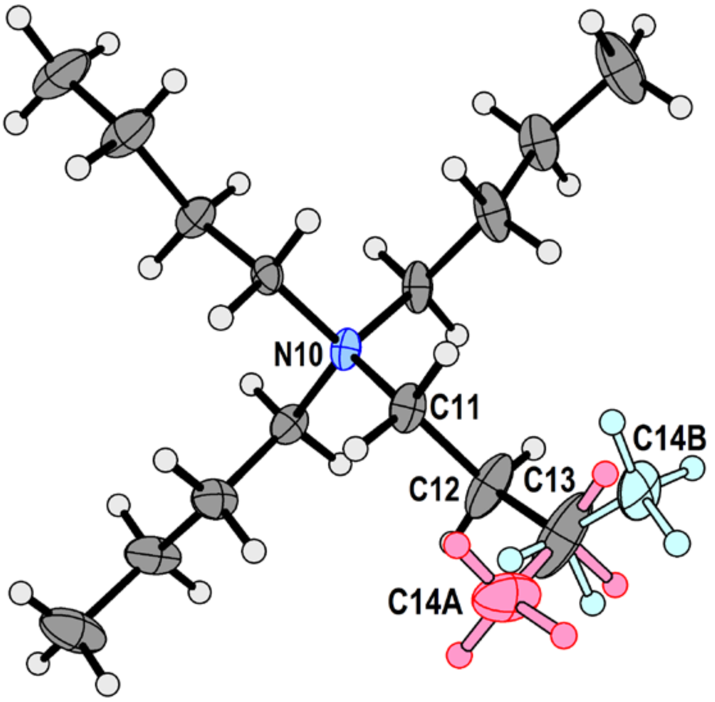

Figure S2.

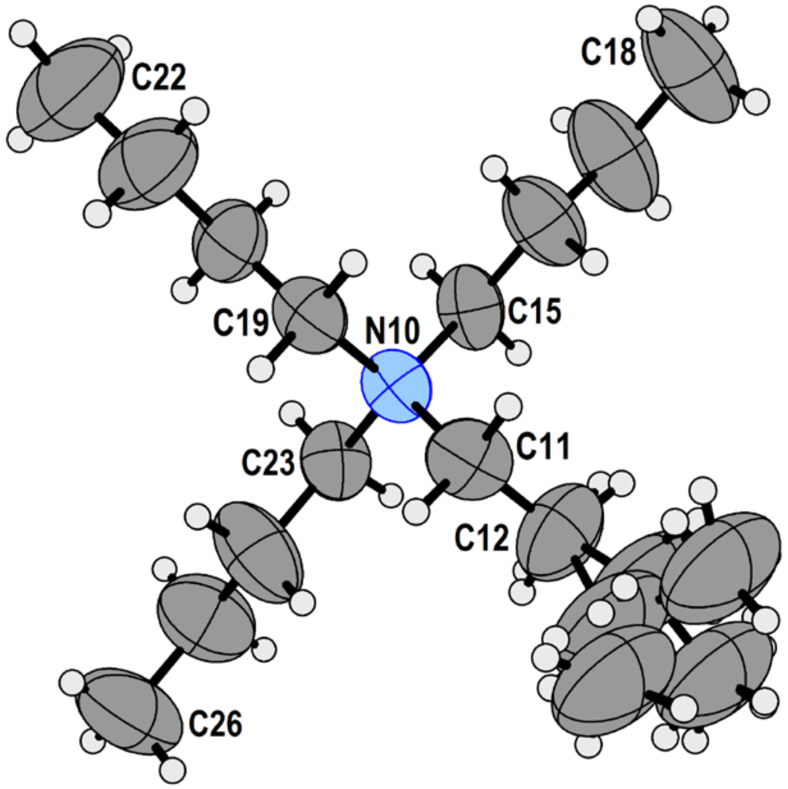

Figure S3.

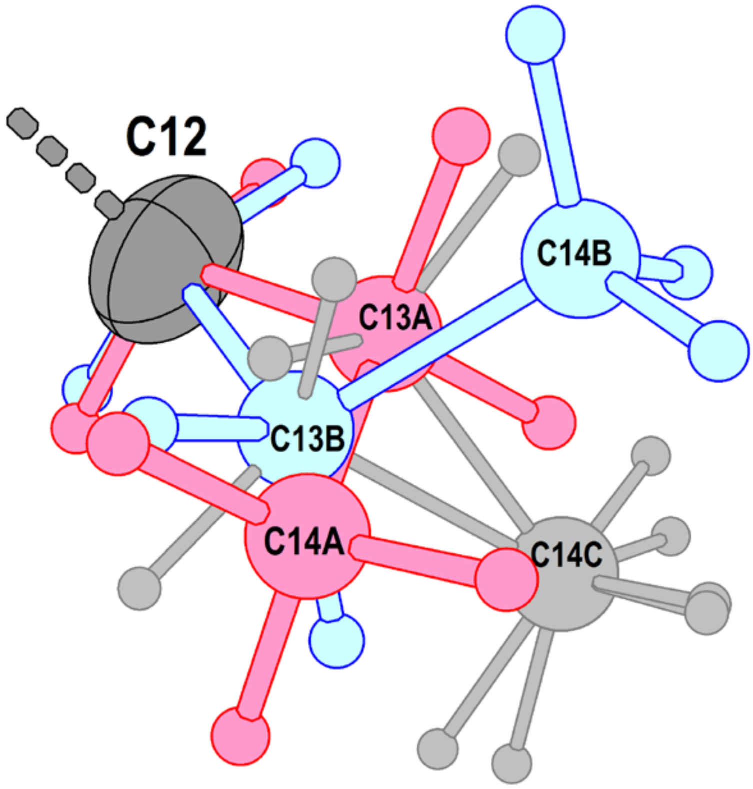

Figure S4.

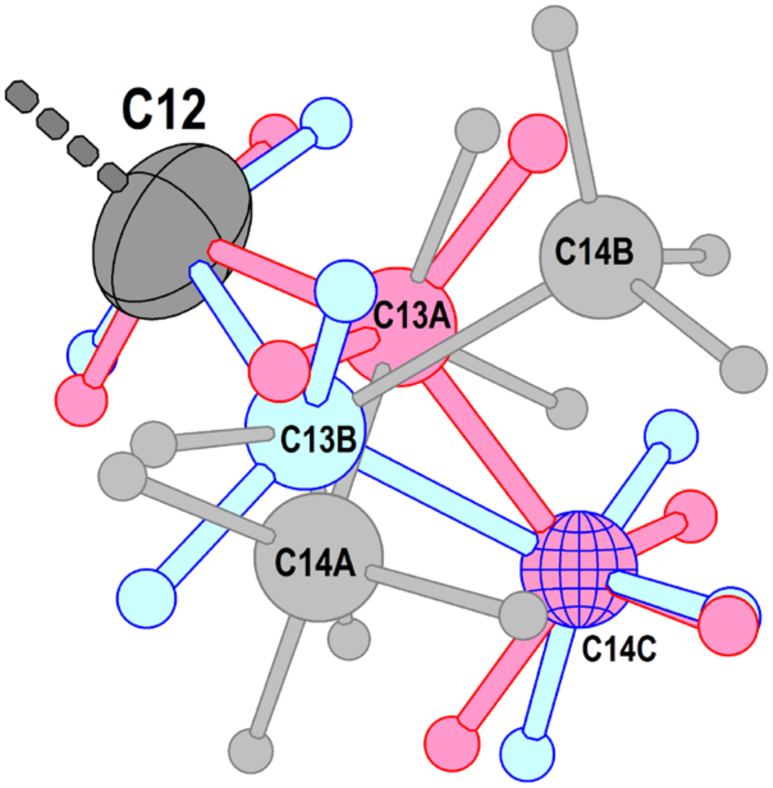

Type	Molecule	Fragment	Classification	No. of hits	Query value	Mean	Std. dev.	| z-score |	| x - mean |	Minimum	Maximum	Median	| d(min) |	Local density
bond	ial12p-l_1	C2 N1	Not unusual (enough hits)	1428	1.374	1.371	0.012	0.326	0.004	1.299	1.452	1.370	0.000	--
bond	ial12p-l_1	C6 N1	Not unusual (enough hits)	18	1.369	1.359	0.011	0.897	0.010	1.342	1.383	1.356	0.001	--
bond	ial12p-l_1	O2 C2	Not unusual (enough hits)	2995	1.222	1.228	0.015	0.374	0.006	1.166	1.520	1.227	0.000	--
bond	ial12p-l_1	C2 N3	Not unusual (enough hits)	1428	1.375	1.371	0.012	0.387	0.005	1.299	1.452	1.370	0.000	--
bond	ial12p-l_1	C4 N3	Not unusual (enough hits)	708	1.385	1.381	0.013	0.324	0.004	1.256	1.419	1.380	0.000	--
bond	ial12p-l_1	O4 C4	Not unusual (enough hits)	1300	1.227	1.241	0.016	0.894	0.014	1.196	1.355	1.240	0.000	--
bond	ial12p-l_1	C5 C4	Not unusual (enough hits)	491	1.448	1.423	0.019	1.346	0.025	1.352	1.464	1.426	0.000	--
bond	ial12p-l_1	C5 C6	Not unusual (enough hits)	24	1.345	1.360	0.011	1.411	0.015	1.339	1.375	1.363	0.002	--
bond	ial12p-l_1	C7 C6	Not unusual (enough hits)	16	1.530	1.481	0.025	1.985	0.049	1.438	1.542	1.483	0.012	--
bond	ial12p-l_1	O7 C7	Not unusual (enough hits)	32	1.250	1.254	0.006	0.734	0.005	1.242	1.275	1.253	0.001	--
bond	ial12p-l_1	O8 C7	Not unusual (enough hits)	32	1.244	1.254	0.006	1.620	0.010	1.242	1.275	1.253	0.002	--
bond	ial12p-l_2	C11 N10	Not unusual (enough hits)	4078	1.516	1.521	0.024	0.234	0.006	1.246	1.915	1.521	0.000	--
bond	ial12p-l_2	C15 N10	Not unusual (enough hits)	4078	1.524	1.521	0.024	0.118	0.003	1.246	1.915	1.521	0.000	--
bond	ial12p-l_2	C19 N10	Not unusual (enough hits)	4078	1.525	1.521	0.024	0.164	0.004	1.246	1.915	1.521	0.000	--
bond	ial12p-l_2	C23 N10	Not unusual (enough hits)	4078	1.524	1.521	0.024	0.122	0.003	1.246	1.915	1.521	0.000	--
bond	ial12p-l_2	C11 C12	Not unusual (enough hits)	4114	1.518	1.512	0.038	0.149	0.006	0.989	1.778	1.516	0.000	--
bond	ial12p-l_2	C13 C12	Not unusual (enough hits)	4212	1.538	1.512	0.057	0.450	0.026	0.951	2.000	1.520	0.000	--
bond	ial12p-l_2	C14A C13	Not unusual (enough hits)	4446	1.408	1.499	0.069	1.329	0.091	0.743	2.085	1.514	0.000	--

bond	ial12p-l_2	C15 C16	Not unusual (enough hits)	4114	1.524	1.512	0.038	0.312	0.012	0.989	1.778	1.516	0.000	--
bond	ial12p-l_2	C17 C16	Not unusual (enough hits)	4212	1.522	1.512	0.057	0.169	0.010	0.951	2.000	1.520	0.000	--
bond	ial12p-l_2	C18 C17	Not unusual (enough hits)	4446	1.520	1.499	0.069	0.307	0.021	0.743	2.085	1.514	0.000	--
bond	ial12p-l_2	C19 C20	Not unusual (enough hits)	4114	1.517	1.512	0.038	0.133	0.005	0.989	1.778	1.516	0.000	--
bond	ial12p-l_2	C21 C20	Not unusual (enough hits)	4212	1.518	1.512	0.057	0.103	0.006	0.951	2.000	1.520	0.000	--
bond	ial12p-l_2	C22 C21	Not unusual (enough hits)	4446	1.520	1.499	0.069	0.307	0.021	0.743	2.085	1.514	0.000	--
bond	ial12p-l_2	C23 C24	Not unusual (enough hits)	4114	1.518	1.512	0.038	0.145	0.006	0.989	1.778	1.516	0.000	--
bond	ial12p-l_2	C25 C24	Not unusual (enough hits)	4212	1.527	1.512	0.057	0.264	0.015	0.951	2.000	1.520	0.000	--
bond	ial12p-l_2	C26 C25	Not unusual (enough hits)	4446	1.524	1.499	0.069	0.363	0.025	0.743	2.085	1.514	0.000	--
angle	ial12p-l_1	O7 C7 C6	No hits	0	116.543	--	--	--	--	--	--	--	--	--
angle	ial12p-l_1	O8 C7 C6	No hits	0	115.525	--	--	--	--	--	--	--	--	--
angle	ial12p-l_1	C6 N1 C2	Not unusual (enough hits)	37	122.843	122.592	1.115	0.225	0.251	119.430	126.873	122.686	0.011	--
angle	ial12p-l_1	O2 C2 N1	Not unusual (enough hits)	1430	123.055	121.890	1.372	0.850	1.166	114.623	129.397	122.097	0.002	--
angle	ial12p-l_1	N3 C2 N1	Not unusual (enough hits)	546	115.073	115.584	1.146	0.445	0.510	111.680	120.111	115.557	0.004	--
angle	ial12p-l_1	O2 C2 N3	Not unusual (enough hits)	1430	121.869	121.890	1.372	0.015	0.021	114.623	129.397	122.097	0.000	--
angle	ial12p-l_1	C4 N3 C2	Not unusual (enough hits)	80	126.061	125.819	0.906	0.268	0.243	123.775	127.201	126.077	0.006	--
angle	ial12p-l_1	O4 C4 N3	Not unusual (enough hits)	708	119.911	119.363	1.239	0.443	0.548	114.543	125.340	119.479	0.000	--
angle	ial12p-l_1	C5 C4 N3	Not unusual (enough hits)	411	114.702	115.159	1.080	0.423	0.457	111.751	118.350	114.989	0.005	--
angle	ial12p-l_1	O4 C4 C5	Not unusual (enough hits)	491	125.386	125.544	1.120	0.141	0.158	120.918	128.433	125.635	0.002	--
angle	ial12p-l_1	C6 C5 C4	Not unusual (enough hits)	39	120.017	120.080	1.157	0.054	0.063	118.559	123.568	119.831	0.030	--

angle	ial12p-l_1	C5 C6 N1	Not unusual (enough hits)	15	120.998	120.844	0.865	0.178	0.154	119.785	123.023	120.575	0.014	--
angle	ial12p-l_1	C7 C6 N1	Not unusual (enough hits)	17	115.491	115.721	0.822	0.280	0.230	114.401	117.095	115.663	0.001	--
angle	ial12p-l_1	C7 C6 C5	Not unusual (enough hits)	15	123.499	123.416	1.147	0.072	0.083	121.820	125.803	123.279	0.115	--
angle	ial12p-l_1	O8 C7 O7	Unusual (enough hits)	16	127.931	124.896	0.744	4.083	3.036	123.030	125.770	125.092	2.161	--
angle	ial12p-l_2	C15 N10 C11	Not unusual (enough hits)	4236	110.828	109.501	3.057	0.434	1.328	71.683	156.520	110.339	0.000	--
angle	ial12p-l_2	C19 N10 C11	Not unusual (enough hits)	4236	106.335	109.501	3.057	1.036	3.166	71.683	156.520	110.339	0.002	--
angle	ial12p-l_2	C23 N10 C11	Not unusual (enough hits)	4236	110.998	109.501	3.057	0.490	1.498	71.683	156.520	110.339	0.000	--
angle	ial12p-l_2	C19 N10 C15	Not unusual (enough hits)	4236	110.025	109.501	3.057	0.172	0.524	71.683	156.520	110.339	0.001	--
angle	ial12p-l_2	C23 N10 C15	Not unusual (enough hits)	4236	107.796	109.501	3.057	0.557	1.704	71.683	156.520	110.339	0.002	--
angle	ial12p-l_2	C23 N10 C19	Not unusual (enough hits)	4236	110.884	109.501	3.057	0.452	1.383	71.683	156.520	110.339	0.001	--
angle	ial12p-l_2	C12 C11 N10	Not unusual (enough hits)	4005	114.968	115.932	3.049	0.316	0.964	87.318	160.053	115.821	0.000	--
angle	ial12p-l_2	C11 C12 C13	Not unusual (enough hits)	4050	109.226	110.660	3.728	0.385	1.434	82.441	169.508	110.390	0.000	--
angle	ial12p-l_2	C14A C13 C12	Not unusual (enough hits)	4246	116.234	113.580	5.905	0.449	2.654	70.885	172.726	113.126	0.007	--
angle	ial12p-l_2	C16 C15 N10	Not unusual (enough hits)	4005	115.361	115.932	3.049	0.187	0.571	87.318	160.053	115.821	0.000	--
angle	ial12p-l_2	C15 C16 C17	Not unusual (enough hits)	4050	110.718	110.660	3.728	0.016	0.058	82.441	169.508	110.390	0.000	--
angle	ial12p-l_2	C18 C17 C16	Not unusual (enough hits)	4246	112.720	113.580	5.905	0.146	0.860	70.885	172.726	113.126	0.000	--
angle	ial12p-l_2	C20 C19 N10	Not unusual (enough hits)	4005	115.267	115.932	3.049	0.218	0.665	87.318	160.053	115.821	0.000	--
angle	ial12p-l_2	C19 C20 C21	Not unusual (enough hits)	4050	110.575	110.660	3.728	0.023	0.085	82.441	169.508	110.390	0.000	--
angle	ial12p-l_2	C22 C21 C20	Not unusual (enough hits)	4246	112.279	113.580	5.905	0.220	1.301	70.885	172.726	113.126	0.002	--
angle	ial12p-	C24 C23 N10	Not unusual (enough	4005	114.513	115.932	3.049	0.465	1.419	87.318	160.053	115.821	0.000	--

	l_2		hits)											
angle	ial12p-l_2	C23 C24 C25	Not unusual (enough hits)	4050	111.289	110.660	3.728	0.169	0.629	82.441	169.508	110.390	0.000	--
angle	ial12p-l_2	C26 C25 C24	Not unusual (enough hits)	4246	111.658	113.580	5.905	0.325	1.922	70.885	172.726	113.126	0.000	--
torsion	ial12p-l_1	O7 C7 C6 N1	Not unusual (enough hits)	40	158.554	--	--	--	--	--	--	--	0.813	0.350
torsion	ial12p-l_1	O8 C7 C6 N1	Not unusual (enough hits)	40	-21.132	--	--	--	--	--	--	--	0.034	0.350
torsion	ial12p-l_1	O7 C7 C6 C5	Not unusual (enough hits)	36	-22.683	--	--	--	--	--	--	--	0.713	0.167
torsion	ial12p-l_1	O8 C7 C6 C5	Not unusual (enough hits)	36	157.631	--	--	--	--	--	--	--	1.948	0.167
torsion	ial12p-l_2	C12 C11 N10 C15	Not unusual (enough hits)	4732	-60.826	--	--	--	--	--	--	--	0.002	0.610
torsion	ial12p-l_2	C12 C11 N10 C19	Not unusual (enough hits)	4732	179.618	--	--	--	--	--	--	--	0.002	0.299
torsion	ial12p-l_2	C12 C11 N10 C23	Not unusual (enough hits)	4732	58.928	--	--	--	--	--	--	--	0.003	0.611
torsion	ial12p-l_2	C16 C15 N10 C11	Not unusual (enough hits)	4732	-52.028	--	--	--	--	--	--	--	0.002	0.466
torsion	ial12p-l_2	C16 C15 N10 C19	Not unusual (enough hits)	4732	65.290	--	--	--	--	--	--	--	0.002	0.521
torsion	ial12p-l_2	C16 C15 N10 C23	Not unusual (enough hits)	4732	-173.682	--	--	--	--	--	--	--	0.006	0.321
torsion	ial12p-l_2	C20 C19 N10 C11	Not unusual (enough hits)	4732	-176.325	--	--	--	--	--	--	--	0.005	0.317
torsion	ial12p-l_2	C20 C19 N10 C15	Not unusual (enough hits)	4732	63.597	--	--	--	--	--	--	--	0.000	0.569
torsion	ial12p-l_2	C20 C19 N10 C23	Not unusual (enough hits)	4732	-55.562	--	--	--	--	--	--	--	0.001	0.576
torsion	ial12p-l_2	C24 C23 N10 C11	Not unusual (enough hits)	4732	55.882	--	--	--	--	--	--	--	0.001	0.582
torsion	ial12p-l_2	C24 C23 N10 C15	Not unusual (enough hits)	4732	177.430	--	--	--	--	--	--	--	0.004	0.313
torsion	ial12p-l_2	C24 C23 N10 C19	Not unusual (enough hits)	4732	-62.080	--	--	--	--	--	--	--	0.003	0.598
torsion	ial12p-l_2	C13 C12 C11 N10	Not unusual (enough hits)	3984	173.303	--	--	--	--	--	--	--	0.002	0.916

torsion	ial12p-l_2	C11 C12 C13 C14A	Not unusual (enough hits)	4215	59.052	--	--	--	--	--	--	--	0.007	0.110
torsion	ial12p-l_2	C17 C16 C15 N10	Not unusual (enough hits)	3984	-167.647	--	--	--	--	--	--	--	0.001	0.755
torsion	ial12p-l_2	C15 C16 C17 C18	Not unusual (enough hits)	4215	-176.639	--	--	--	--	--	--	--	0.001	0.697
torsion	ial12p-l_2	C21 C20 C19 N10	Not unusual (enough hits)	3984	-173.009	--	--	--	--	--	--	--	0.000	0.919
torsion	ial12p-l_2	C19 C20 C21 C22	Not unusual (enough hits)	4215	177.663	--	--	--	--	--	--	--	0.000	0.688
torsion	ial12p-l_2	C25 C24 C23 N10	Not unusual (enough hits)	3984	170.921	--	--	--	--	--	--	--	0.000	0.943
torsion	ial12p-l_2	C23 C24 C25 C26	Not unusual (enough hits)	4215	-178.309	--	--	--	--	--	--	--	0.002	0.682

Type	Molecule	Fragment	Classification	No. of hits	Query value	Mean	Std. dev.	| z-score |	| x - mean |	Minimum	Maximum	Median	| d(min) |	Local density
bond	est4l_1	C2 N1	Not unusual (enough hits)	1428	1.365	1.371	0.012	0.483	0.006	1.299	1.452	1.370	0.000	--
bond	est4l_1	C6 N1	Not unusual (enough hits)	18	1.367	1.359	0.011	0.711	0.008	1.342	1.383	1.356	0.001	--
bond	est4l_1	O2 C2	Not unusual (enough hits)	2995	1.215	1.228	0.015	0.846	0.013	1.166	1.520	1.227	0.000	--
bond	est4l_1	C2 N3	Not unusual (enough hits)	1428	1.369	1.371	0.012	0.124	0.001	1.299	1.452	1.370	0.000	--
bond	est4l_1	C4 N3	Not unusual (enough hits)	708	1.383	1.381	0.013	0.199	0.003	1.256	1.419	1.380	0.000	--
bond	est4l_1	O4 C4	Not unusual (enough hits)	1300	1.225	1.241	0.016	1.016	0.016	1.196	1.355	1.240	0.000	--
bond	est4l_1	C5 C4	Not unusual (enough hits)	491	1.431	1.423	0.019	0.405	0.008	1.352	1.464	1.426	0.000	--
bond	est4l_1	C7 C6	Not unusual (enough hits)	16	1.522	1.481	0.025	1.665	0.041	1.438	1.542	1.483	0.010	--
bond	est4l_1	C5 C6	Unusual (enough hits)	24	1.327	1.360	0.011	3.065	0.034	1.339	1.375	1.363	0.012	--
bond	est4l_1	O7 C7	Unusual (enough hits)	32	1.235	1.254	0.006	3.047	0.019	1.242	1.275	1.253	0.007	--
bond	est4l_1	O8 C7	Unusual (enough hits)	32	1.224	1.254	0.006	4.820	0.031	1.242	1.275	1.253	0.019	--
bond	est4l_2	C15 N10	Not unusual (enough hits)	4078	1.519	1.521	0.024	0.098	0.002	1.246	1.915	1.521	0.000	--
bond	est4l_2	C19 N10	Not unusual (enough hits)	4078	1.509	1.521	0.024	0.524	0.012	1.246	1.915	1.521	0.000	--
bond	est4l_2	C23 N10	Not unusual (enough hits)	4078	1.525	1.521	0.024	0.168	0.004	1.246	1.915	1.521	0.000	--
bond	est4l_2	C15 C16	Not unusual (enough hits)	4114	1.521	1.512	0.038	0.227	0.009	0.989	1.778	1.516	0.000	--
bond	est4l_2	C17 C16	Not unusual (enough hits)	4212	1.444	1.512	0.057	1.209	0.069	0.951	2.000	1.520	0.000	--
bond	est4l_2	C18 C17	Not unusual (enough hits)	4446	1.485	1.499	0.069	0.197	0.014	0.743	2.085	1.514	0.000	--
bond	est4l_2	C19 C20	Not unusual (enough hits)	4114	1.504	1.512	0.038	0.221	0.008	0.989	1.778	1.516	0.000	--
bond	est4l_2	C21 C20	Not unusual (enough hits)	4212	1.489	1.512	0.057	0.410	0.023	0.951	2.000	1.520	0.000	--
bond	est4l_2	C22 C21	Not unusual (enough	4446	1.504	1.499	0.069	0.078	0.005	0.743	2.085	1.514	0.000	--

			hits)											
bond	est4l_2	C23 C24	Not unusual (enough hits)	4114	1.532	1.512	0.038	0.515	0.020	0.989	1.778	1.516	0.000	--
bond	est4l_2	C25 C24	Not unusual (enough hits)	4212	1.416	1.512	0.057	1.697	0.097	0.951	2.000	1.520	0.000	--
bond	est4l_2	C26 C25	Not unusual (enough hits)	4446	1.504	1.499	0.069	0.070	0.005	0.743	2.085	1.514	0.000	--
angle	est4l_1	O7 C7 C6	No hits	0	116.313	--	--	--	--	--	--	--	--	--
angle	est4l_1	O8 C7 C6	No hits	0	116.170	--	--	--	--	--	--	--	--	--
angle	est4l_1	C6 N1 C2	Not unusual (enough hits)	37	124.008	122.592	1.115	1.270	1.416	119.430	126.873	122.686	0.037	--
angle	est4l_1	O2 C2 N1	Not unusual (enough hits)	1430	124.065	121.890	1.372	1.586	2.176	114.623	129.397	122.097	0.010	--
angle	est4l_1	N3 C2 N1	Not unusual (enough hits)	546	113.971	115.584	1.146	1.406	1.612	111.680	120.111	115.557	0.001	--
angle	est4l_1	O2 C2 N3	Not unusual (enough hits)	1430	121.961	121.890	1.372	0.052	0.071	114.623	129.397	122.097	0.002	--
angle	est4l_1	C4 N3 C2	Not unusual (enough hits)	80	126.241	125.819	0.906	0.465	0.422	123.775	127.201	126.077	0.002	--
angle	est4l_1	O4 C4 N3	Not unusual (enough hits)	708	119.441	119.363	1.239	0.063	0.078	114.543	125.340	119.479	0.000	--
angle	est4l_1	C5 C4 N3	Not unusual (enough hits)	411	114.568	115.159	1.080	0.547	0.591	111.751	118.350	114.989	0.001	--
angle	est4l_1	O4 C4 C5	Not unusual (enough hits)	491	125.990	125.544	1.120	0.398	0.446	120.918	128.433	125.635	0.000	--
angle	est4l_1	C6 C5 C4	Not unusual (enough hits)	39	120.772	120.080	1.157	0.599	0.692	118.559	123.568	119.831	0.088	--
angle	est4l_1	C5 C6 N1	Not unusual (enough hits)	15	120.107	120.844	0.865	0.852	0.737	119.785	123.023	120.575	0.063	--
angle	est4l_1	C7 C6 N1	Not unusual (enough hits)	17	115.500	115.721	0.822	0.269	0.221	114.401	117.095	115.663	0.010	--
angle	est4l_1	C7 C6 C5	Not unusual (enough hits)	15	124.386	123.416	1.147	0.846	0.970	121.820	125.803	123.279	0.190	--
angle	est4l_1	O8 C7 O7	Unusual (enough hits)	16	127.508	124.896	0.744	3.514	2.613	123.030	125.770	125.092	1.738	--
angle	est4l_2	C19 N10 C15	Not unusual (enough hits)	4236	109.834	109.501	3.057	0.109	0.333	71.683	156.520	110.339	0.002	--
angle	est4l_2	C23 N10 C15	Not unusual (enough hits)	4236	106.916	109.501	3.057	0.846	2.585	71.683	156.520	110.339	0.001	--
angle	est4l_2	C23 N10 C19	Not unusual (enough	4236	111.217	109.501	3.057	0.561	1.716	71.683	156.520	110.339	0.001	--

			hits)											
angle	est4l_2	C16 C15 N10	Not unusual (enough hits)	4005	115.615	115.932	3.049	0.104	0.316	87.318	160.053	115.821	0.001	--
angle	est4l_2	C15 C16 C17	Not unusual (enough hits)	4050	113.239	110.660	3.728	0.692	2.579	82.441	169.508	110.390	0.002	--
angle	est4l_2	C18 C17 C16	Not unusual (enough hits)	4246	116.470	113.580	5.905	0.489	2.890	70.885	172.726	113.126	0.003	--
angle	est4l_2	C20 C19 N10	Not unusual (enough hits)	4005	116.012	115.932	3.049	0.026	0.080	87.318	160.053	115.821	0.000	--
angle	est4l_2	C19 C20 C21	Not unusual (enough hits)	4050	111.860	110.660	3.728	0.322	1.200	82.441	169.508	110.390	0.003	--
angle	est4l_2	C22 C21 C20	Not unusual (enough hits)	4246	113.537	113.580	5.905	0.007	0.043	70.885	172.726	113.126	0.001	--
angle	est4l_2	C24 C23 N10	Not unusual (enough hits)	4005	114.355	115.932	3.049	0.517	1.577	87.318	160.053	115.821	0.000	--
angle	est4l_2	C23 C24 C25	Not unusual (enough hits)	4050	114.761	110.660	3.728	1.100	4.101	82.441	169.508	110.390	0.015	--
angle	est4l_2	C26 C25 C24	Not unusual (enough hits)	4246	115.327	113.580	5.905	0.296	1.747	70.885	172.726	113.126	0.000	--
torsion	est4l_1	O7 C7 C6 N1	Not unusual (enough hits)	40	161.730	--	--	--	--	--	--	--	0.040	0.350
torsion	est4l_1	O8 C7 C6 N1	Not unusual (enough hits)	40	-19.239	--	--	--	--	--	--	--	1.418	0.350
torsion	est4l_1	O7 C7 C6 C5	Not unusual (enough hits)	36	-19.222	--	--	--	--	--	--	--	0.018	0.389
torsion	est4l_1	O8 C7 C6 C5	Not unusual (enough hits)	36	159.809	--	--	--	--	--	--	--	0.090	0.333
torsion	est4l_2	C16 C15 N10 C19	Not unusual (enough hits)	4732	60.014	--	--	--	--	--	--	--	0.004	0.613
torsion	est4l_2	C16 C15 N10 C23	Not unusual (enough hits)	4732	-179.201	--	--	--	--	--	--	--	0.000	0.302
torsion	est4l_2	C20 C19 N10 C15	Not unusual (enough hits)	4732	60.798	--	--	--	--	--	--	--	0.000	0.611
torsion	est4l_2	C20 C19 N10 C23	Not unusual (enough hits)	4732	-57.355	--	--	--	--	--	--	--	0.003	0.604
torsion	est4l_2	C24 C23 N10 C15	Not unusual (enough hits)	4732	177.352	--	--	--	--	--	--	--	0.001	0.313
torsion	est4l_2	C24 C23 N10 C19	Not unusual (enough hits)	4732	-62.748	--	--	--	--	--	--	--	0.000	0.588

torsion est4l_2	C17 C16 C15 N10	Not unusual (enough hits)	3984	177.527	--	--	--	--	--	--	--	0.001	0.825
torsion est4l_2	C15 C16 C17 C18	Not unusual (enough hits)	4215	179.726	--	--	--	--	--	--	--	0.000	0.661
torsion est4l_2	C21 C20 C19 N10	Not unusual (enough hits)	3984	-176.180	--	--	--	--	--	--	--	0.002	0.860
torsion est4l_2	C19 C20 C21 C22	Not unusual (enough hits)	4215	179.217	--	--	--	--	--	--	--	0.001	0.670
torsion est4l_2	C25 C24 C23 N10	Not unusual (enough hits)	3984	-179.338	--	--	--	--	--	--	--	0.001	0.762
torsion est4l_2	C23 C24 C25 C26	Not unusual (enough hits)	4215	179.249	--	--	--	--	--	--	--	0.000	0.670
